# Explicating collegiality and change management in neoliberalism during the dynamics of higher education institutions: : A Systematic Literature Review using PRISMA Checklist

**DOI:** 10.12688/f1000research.146044.3

**Published:** 2025-03-07

**Authors:** Makna Ani Marlia, Rahmi Fahmy, Hendra Lukito, Donard Games

**Affiliations:** 1 Department of Management, Faculty of Economics and Business, Universitas Andalas, Padang, West Sumatra, 25000, Indonesia

**Keywords:** change management, collegiality, neoliberalism, NPM, managerialism, higher education

## Abstract

**Background:**

The neoliberal approach to higher education management has significantly transformed collegiality and university governance. This study identifies and analyzes how and where these changes have occurred, as well as their impact on the efficiency and competitiveness of universities. It addresses existing research gaps and provides new insights for adaptation strategies.

**Methods:**

We selected all retrieved sources based on the keywords and analyzed all the documents we obtained. This study obtained data from Scopus retrieved on October 27, 2023, using the following keywords: (TITLE-ABS-KEY (“collegiality”) OR TITLE-ABS-KEY (“change management”) OR TITLE-ABS-KEY (“neolibelism”)) AND TITLE-ABS-KEY (“higher education”). This study utilized bibliometric analysis to ensure a structured review of the literature on collegiality, change management, and neoliberalism in higher education.

**Results:**

The findings show that organizational management, leadership, education, technology, curriculum, innovation, organizational change, decision-making, and human beings are significant trajectories of neoliberalism in higher education.

**Conclusions:**

This study offers other constructs for accelerating leadership success in higher education. This relates to how change leadership can navigate changes resulting from neoliberal ideology.

## Introduction

Globalization and neoliberalism in higher education create different pressures and demands for reform. Adopting neoliberal behavior and ideology focuses on the importance of market dynamics in social exchange throughout university activities. Higher education has shaped changes in organizational practices, processes, and culture ( Ajayan & Balasubramanian, 2020; Croucher & Lacy, 2022; Gaus, Basri, Thamrin, & Ritonga, 2020; Hamlin & Patel, 2017). Traditionally, before the 1980s, universities were established for public and collegial management aimed at public welfare, which was considered the best way to manage all the unique attributes of universities. Before neoliberalism, universities were influenced by the ideology of managerialism, which radically uprooted this idea ( Fleming, 2020). The adoption of neoliberal ideology in the form of New Public Management (NPM) or New Managerialism has shifted the traditional approach to what is said to be a corporate or business approach ( Ajayan & Balasubramanian, 2020; Fleming, 2020; Martin-Sardesai, Guthrie, Tooley, & Chaplin, 2019).

Osborne and Gaebler (1992) developed the NPM in the context of the United States, which has promoted the principles of business in Higher Education through value for money, quality assurance, monitoring, evaluation, auditing, and accountability ( Ajayan & Balasubramanian, 2020; Gaus et al., 2020; Gaus, Tang, & Akil, 2019; Gaus & Hall, 2015). Integrating the concept of corporatization or commercial practices into higher education and academia and using performance-based metrics indicates government control ( Gaus & Hall, 2016) and coercion disguised through technological policies ( Foucault, 1982). This undermines academic independence and autonomy, resulting in the prioritization of financial interests in public service administration, commonly referred to as a “money culture”.

Despite the corrosive effects of the NPM agenda on higher education and the work of academia, it continues to evolve in higher education worldwide ( Christopher, 2012a; Haake, 2009; Hossan, 2015; Middlehurst, 2004; Yokoyama, 2006), including Indonesia ( Gaus, 2019b; Gaus et al., 2020, 2019; Gaus & Hall, 2015, 2016). The governments of Malaysia and Thailand strongly believe in the doctrine of neo-liberalism, believing that implementing market concepts and strategies would enhance service delivery and foster good governance in the public sector ( Gaus & Tang, 2023; Taib & Abdullah, 2015). These changes affected each university’s mission, values, and overall operations at different levels of change.

Some researchers have discussed the application of NPM/managerialism in higher education, such as Mok (1999), in his study of higher education sectors in Hong Kong, stating that implementing managerialism has led organizations to adopt a more “customer-oriented” approach and treat consumers. Kalfa and Taksa (2016) found that institutions that follow the practice of managerialism focus on developing robust relationships with stakeholders, such as industries that expect graduates to have the skills to obtain employment. Thus, managerialism requires universities to be more entrepreneurial in terms of having alternative earnings mechanisms ( Ajayan & Balasubramanian, 2020). However, Nickson (2014) lamented that colleges may be able to sacrifice academic freedom and collegiality to obtain alternative sources of income. Gaus and Tang (2023) espoused NPM from the application of neoliberal ideology with its economic rationality leading to the corporatization of higher education has preserved the business lexicon, such as individualism, competence, effectiveness, and efficiency, where individual and personal interests take precedence over the interests of society.

This is in line with Fleming’s (2020) expressed as The Dark Academia, which revealed that the transformation resulting from neoliberal currents had changed universities to become more corporative, more commercial, and universities such as Edu Factory, which produce mass and make students as consumers who need to be satisfied and served, while lecturers become factors of production and isolated from the ideal world. Where the lecturer profession is a lifestyle, not everyone can enter it. Academics are highly trained specialists who have dedicated themselves and time to the grueling study of their chosen discipline. No one in the organization, including the university’s line manager, is more knowledgeable about their teaching and research fields. A simple top-down hierarchy does not work in this setting and becomes a barrier. Unlike manual workers, who have to produce a certain amount that can be quantified, academic work is abstract and cannot be driven by performance incentives like factories, but academics carry out the profession in this sector wholeheartedly.

The government is closely associated with neoliberalism in its role as a regulatory authority. This ideology seeks to address inefficiencies in the public sector by implementing corporate practice. The aim is to make universities more efficient, productive, customer-oriented, and accountable ( Ajayan & Balasubramanian, 2020). The World Bank, UNESCO, and OECD endorse the evolving perspective that education may be treated as a tradable asset with the potential to generate lucrative revenue ( Lynch, 2015). Universities worldwide face the challenge of adapting to the growing number of “business-like” Higher Education institutions. This necessitates a shift towards a more entrepreneurial approach, focusing on achieving greater efficiency and improving overall quality ( Robertson, 2010). In addition, the rise of managerialism/new Public Management (NPM) in the higher education sector is motivated by the need to showcase academic standards (quality assurance/QA) and research output to secure government and industry funding, predominantly contingent on performance. Additionally, there is a need to enhance an institution’s reputation to attract both local and international students ( Ajayan & Balasubramanian, 2020).

Various phenomena that arise due to the adoption of the ideology of neoliberalism also seem to cause other corrosive effects, especially pressure from various stakeholders in higher education; the primary sources of pressure on universities are students, the government, the business world, and local communities. This has resulted in universities having to seriously examine their management and governance. Carbone et al. (2019) found that higher education that nurtures the value of collegiality tends to be more successful in managing change by maintaining the quality of the education. However, the study involved only one institution of higher education and the findings may not be generalizable to other contexts. On the other hand, Brown and Edmunds (2020) showed that neoliberalism often encourages higher education to sacrifice collegiality for efficiency and competition. This can lead to tension between lecturers and university management, which may affect the quality of the education.

Furthermore, in Europe and the United States, research indicates that these changes have led to a decline in academic collegiality, where relationships among academics have become more competitive and less collaborative ( Lawless & Chen, 2016). In Indonesia, a similar phenomenon is observed at Gadjah Mada University and the University of Indonesia, where studies show that the adoption of neoliberal principles has diminished the spirit of collegiality among academics ( Sahoo, 2023). This reflects a global trend in which universities are transitioning from public missions to market-oriented entities, potentially undermining academic integrity and collaboration ( Tyson et al., 2020).

These changes also impact the quality of governance in higher education institutions. A study in Saudi Arabia revealed that less than one-third of the surveyed faculty members received training in corporate governance practices, indicating a lack of preparedness to meet the demands of neoliberalism in education ( Omar et al., 2022). In Indonesia, this is evident in how higher education policies are increasingly influenced by market logic, leading to a reduction in support for academic collaboration and an increased focus on measurable outcomes ( Kabir, 2013). Thus, the adoption of neoliberalism not only alters the management of universities but also transforms the social and professional relationships within them, which in turn affects the quality of education provided.

Thus, previous governance models based on collegiality may not be fully sustained considering customer pressure regarding business-like expectation responses in dynamic settings ( Davies, Hides, & Casey, 2001). The rationale for the review in the context of existing knowledge is to address the gap in understanding how neoliberalism influences collegiality and change management in higher education institutions. Neoliberal ideology has significantly impacted the governance and operation of universities, leading to shifts in power dynamics, decision-making processes, and organizational structures. By examining the existing literature on this topic, the review aims to provide insights into universities’ challenges and opportunities in adapting to neoliberal reforms. This paper discusses the lack of references that comprehensively describe the changes that occur in universities owing to the ideology of neoliberalism. This research also discusses how a university maintains its function and identity when the great wave of neoliberalism massively erodes the collegiality that was the original philosophy of the university, through a case study of the impact of neoliberalism ideology on higher education. This study also provides a comprehensive framework for changes in higher education management and governance. This research provides three research questions as follows:

• How does neoliberalism erode collegiality in higher education?
• How does the ideology of neoliberalism give a pattern to change in higher education?
• What kind of management and governance can increase the competitiveness of universities in facing the challenges of the neoliberal environment?

## Literature review

Change is identified as a shift in organizational behavior from one form to another. Change management has been identified as continuously upgrading the supervision, structure, and service to meet customer needs, either internal or external ( Akinbode & Shuhumi, 2018; Almanei, Salonitis, & Tsinopoulos, 2018). Organizational change is necessary when organizations are no longer in harmony with their outward setting and existence is vulnerable ( Price & Chahal, 2006; Oreg, Vakola, & Armenakis, 2011). However, organizations continually encounter fierce struggles under pressure that require them to adjust their strategies, technologies, and other processes to survive ( Almanei et al., 2018). Change is a steady, continuous process, and not just a situation that occurs once ( Almanei et al., 2018). Change management is a discipline that provides the transition among individuals, teams, or entire managed organizations to lead and guide the process to the desired state by solving various types of resistance ( Almanei et al., 2018).

Based on several theories about change management that have been explained by Lewin’s theory ( Lewin, 1947) is the most critical concept of change methods development ( Aldulaimi & Abdeldayem, 2020) and is the theoretical basis for modern change models ( Vlachopoulos, 2021) which are built from the first three stages of change, unfreezing, which is the process of awareness about the need or need to change. Second, changing/moving is a step action to strengthen driving forces or reduce resistance, and third, refreezing is bringing the organization back to a new equilibrium, better known as a new dynamic equilibrium, and researchers make this theory a grand theory of change management in this study.

**Figure 1.**
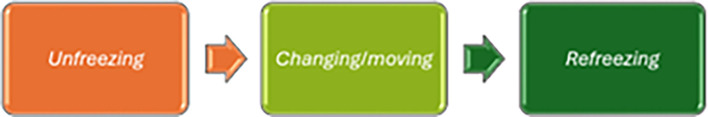
Concept Map of Changes in Universities ( Lewin, 1951).

Universities have undergone significant changes in governance and management. This has been well-documented in various studies ( Bolden, Petrov, & Gosling, 2009; Clegg & McAuley, 2005; Enders, De Boer, & Leisyte, 2008; Hamlin & Patel, 2017; Smith, 2005; Vuori, 2015). Traditionally, the College is governed by policies that administrative leaders manage. However, changes driven by various stakeholders make today’s universities manage business management ( Davies et al., 2001; Gaus et al., 2020; Gaus & Tang, 2023). These changes have led to academic field transformation in universities into business units run by management, focusing on targets such as companies that work under tight budget limits ( Fleming, 2020; Waring, 2017). Based on the literature, a comprehensive analysis of the central changes that occur in universities is presented as a concept map of changes in universities as shown in Figure 1.

## Methods

### The design of the study

This research is quantitative with a systematic approach to literature review using bibliometric analysis, and Biblioshiny software version 4.0 is used to analyze data. Bibliometric analysis is used with the consideration that bibliometrics studies can be used to see trends in a field of study and the productivity of journals/authors and is a combination of mathematical models and statistical models ( Gumus, Bellibas, Esen, & Gumus, 2018). This method allows researchers to examine abstracts, keywords, and references to specific field studies to reveal their authors, countries, journals, and institutions and can generate scientific collaboration between researchers worldwide. Despite the increase in bibliometric interest in various academic fields, studies using this method are still interesting to explore further in the field of study of each researcher.

### Data collection and analysis

**Figure 2.**
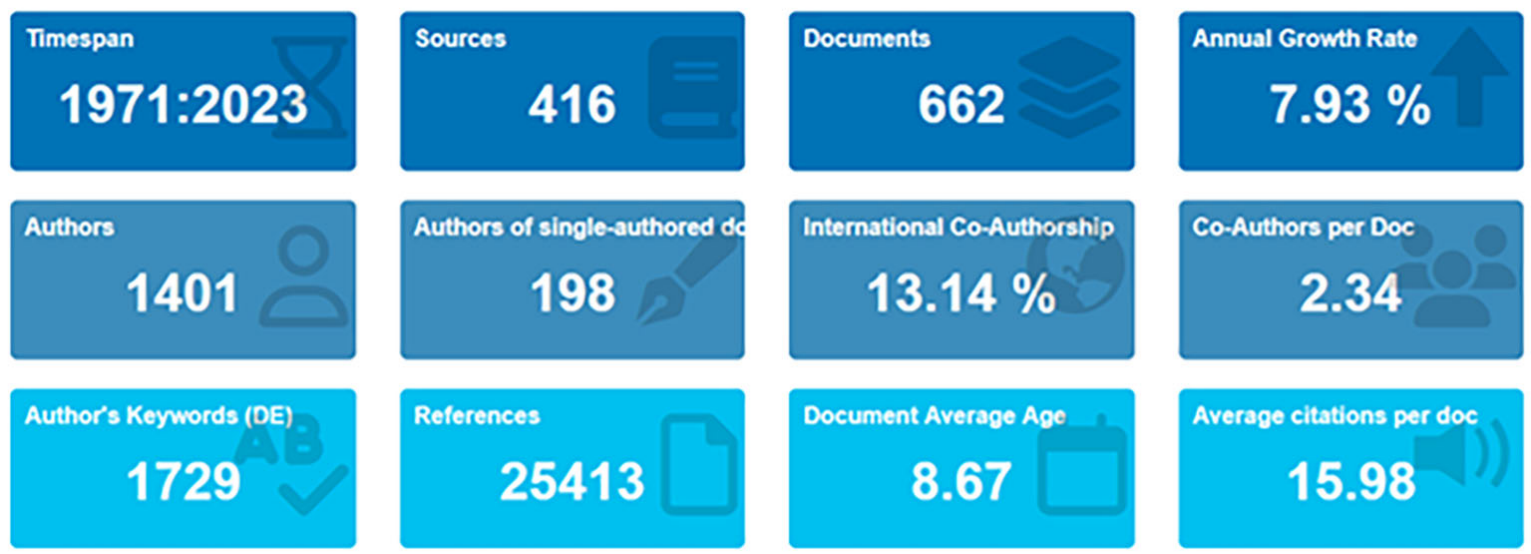
Primary information.

We selected all retrieved sources based on the keywords and analyzed all the documents we obtained. This study obtained data from Scopus retrieved on October 27, 2023, using the following keywords: (TITLE-ABS-KEY (“collegiality”) OR TITLE-ABS-KEY (“change management”) OR TITLE-ABS-KEY (“neolibelism”)) AND TITLE-ABS-KEY (“higher education”). After that, 416 sources were retrieved in Scopus Database within 1971 to 2023 publication year, then 662 documents were obtained ( Marlia *et al.*, 2024). In the primary information as shown in Figure 2 and Table 1, it can be seen that there are 662 documents consists of 198 documents that only have single authors with the number of authors consisting of 1401 authors. Of the 1401 authors, approximately 13.14% were listed as international writers; each author wrote an average of 234 articles.

**Table 1.** Primary information.

Description	Results
Data on primary information	
Timespan	1971:2023
Sources (Journals, Books, etc.)	416
Documents	662
Annual growth rate %	7,93
Document average age	8,67
Average citations per doc	15,98
References	25413
Contents	
Keywords plus (ID)	1021
Author’s keywords (DE)	1729
Authors	
Authors	1401
Authors of single-authored docs	198
Collaboration	
Single-authored docs	213
Co-Authors per doc	2,34
International co-authorships %	13,14
Types	
article	446
book	22
book chapter	68
conference paper	85
conference review	2
editorial	2
note	4
review	33

Table 1 shows that, based on document type, most (446 documents) are articles, and the rest are books, book chapters, conference papers, editorials, notes, and reviews.

We visually display the results of individual studies and syntheses. Tabular presentation of data from individual studies, including author names, publication years, affiliations, and key findings related to collegiality, change management, and neoliberalism in higher education. Comparative tables to highlight differences or similarities between studies in terms of methodologies, results, and implications. Visualization of keyword networks to show relationships between key concepts such as higher education, change management, collegiality, and neoliberalism. Visual representation of the frequency of words or keywords related to collegiality, change management, and neoliberalism in higher education, with the size of each word indicating its occurrence in the articles. Displaying the top 100 words based on the word cloud found in articles, with the size of each word representing the number of occurrences in the article titles. Visual representation of changes in universities influenced by neoliberalism, based on Lewin’s model ( Lewin, 1951). By utilizing these methods for tabulating and visually displaying results, we could present a comprehensive overview of the individual studies and syntheses related to collegiality, change management, and neoliberalism in higher education, facilitating a better understanding of the complex dynamics within this academic domain.

Based on Lewin’s model of change in universities, concept mapping was used to illustrate the transformation processes influenced by neoliberalism in higher education. This method provides a structured framework for understanding the changes occurring in educational organizations and the adoption of new practices. Bibliometric analysis was used to analyze the productivity of authors, journals, and institutions in collegiality, change management, and neoliberalism in higher education. This method helps identify trends, collaborations, and impact within the academic community, providing quantitative insights into research output. We also assessed the quality of individual studies through tools like the PRISMA Checklist ( Marlia et al., 2024) or risk of bias assessment can help identify sources of heterogeneity related to study design, methodology, or reporting. By evaluating study quality, we can determine the reliability and validity of results and assess the impact of study quality on overall findings.

### Ethical considerations

We also involved two fresh professors for data interpretation review. They confirmed our data regarding the Scopus sources and our data interpretation to Biblioshiny output. This study is conducted under ethical approval from Institutional Review Board of Andalas University through ethical approval number: 314/UN16.05/S3.M/2023 on October 2 ^nd^, 2023.

## Results

The findings of this research are structured to directly address the established research questions. First, the discussion focuses on how neoliberalism erodes collegiality within universities, presenting supporting data for each finding through relevant visualizations. Subsequently, the patterns of change identified in response to neoliberal pressures are explained. Finally, the emerging management and governance strategies derived from the data are outlined as responses to the challenges posed by neoliberalism.

Data analysis with bibliometrics on the trend of research development is presented in this study through several visualizations, such as publication productivity from year to year, from publication in 1971 to 2023, and the results of analysis from the world cloud and Tree Map as a whole present trends in researchers’ discussions about the relationships between collegiality, change management, and neoliberalism in higher education. Researchers in this case also criticize the field of study, which is mostly discussed based on the results of this study. Researchers expect to make significant contributions to the literature. We believe that university leaders must prioritize some issues related to the impact of neoliberal ideology that must be executed with change management in universities without obscuring the initial philosophy or nature of higher education. The following are the results and visualizations of the data analysis using bibliometrics:

### Publication productivity

**Figure 3.**
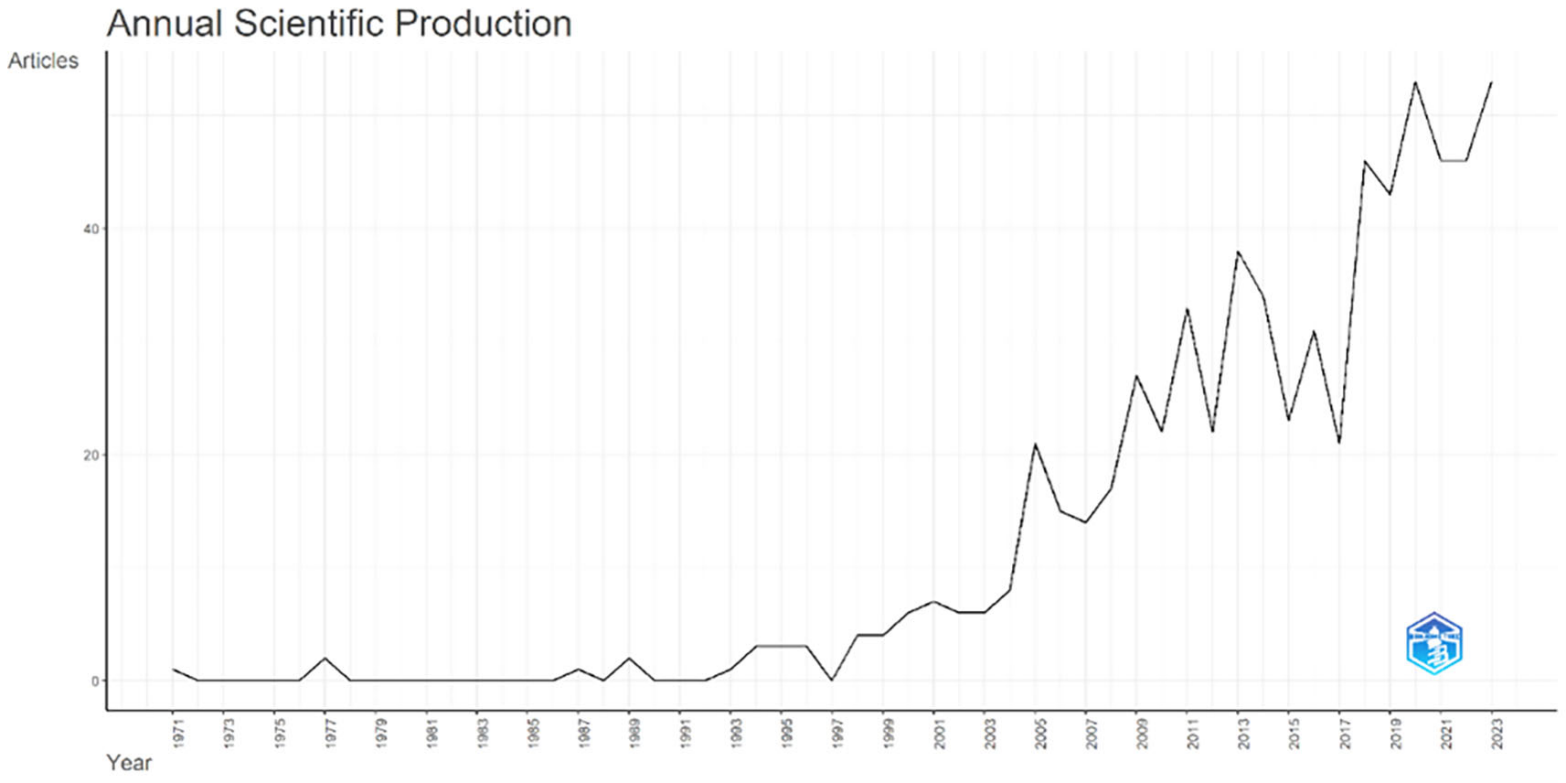
Annual scientific production.

**Figure 4.**
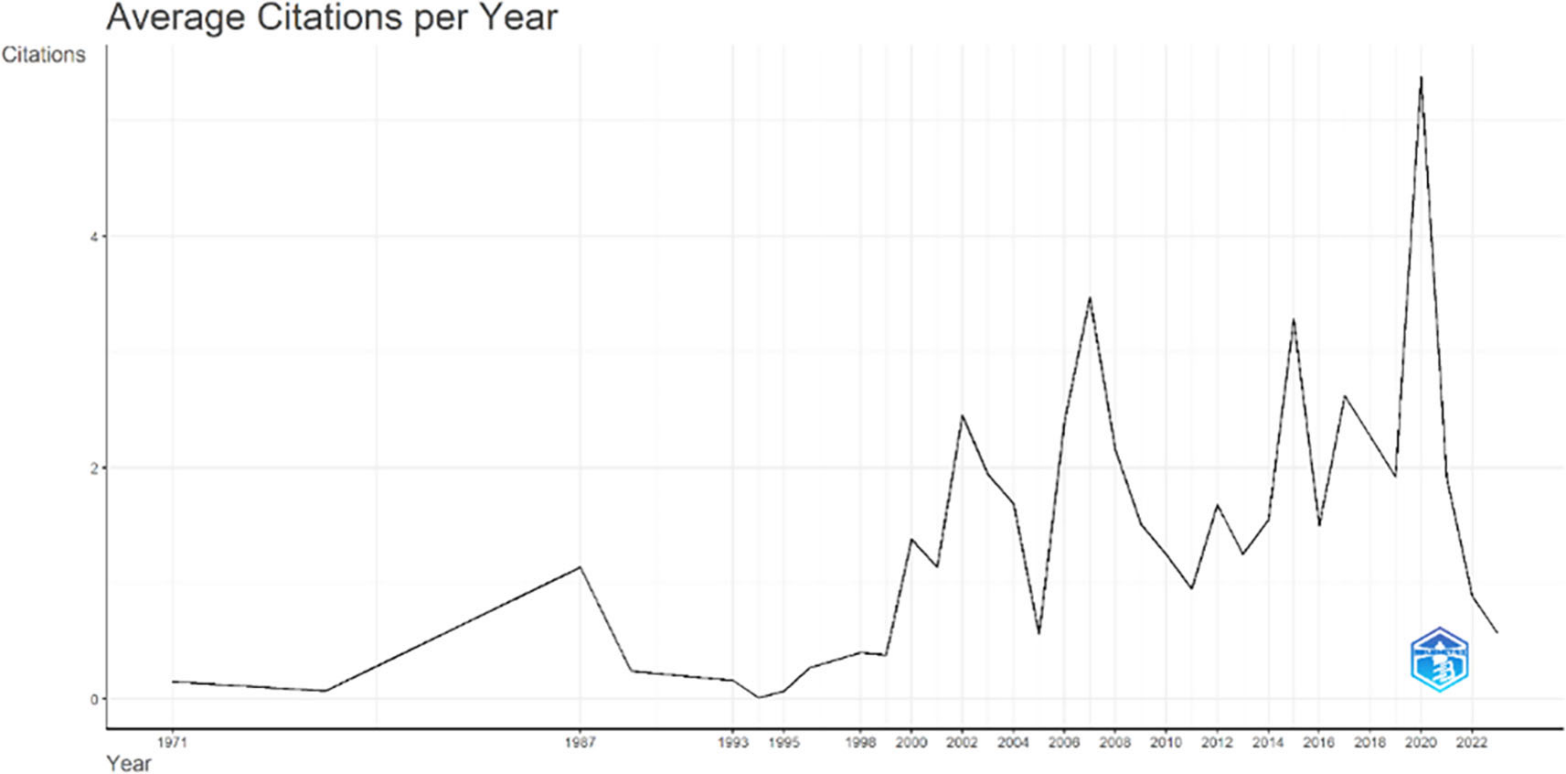
Average article citation per year.

Annual Scientific Production monitors the growth of scientific work production over time. In Figure 3, it can be seen that there was an increase in articles from year to year, with a significant spike occurring from 2017 to 2018. This means that there has been an increase in productivity related to studies that discuss the relationships between collegiality, change management, and neoliberalism in higher education. This productivity is also supported by the high average number of citations per year, which has also increased. This is illustrated in Figure 4.

**Figure 5.**
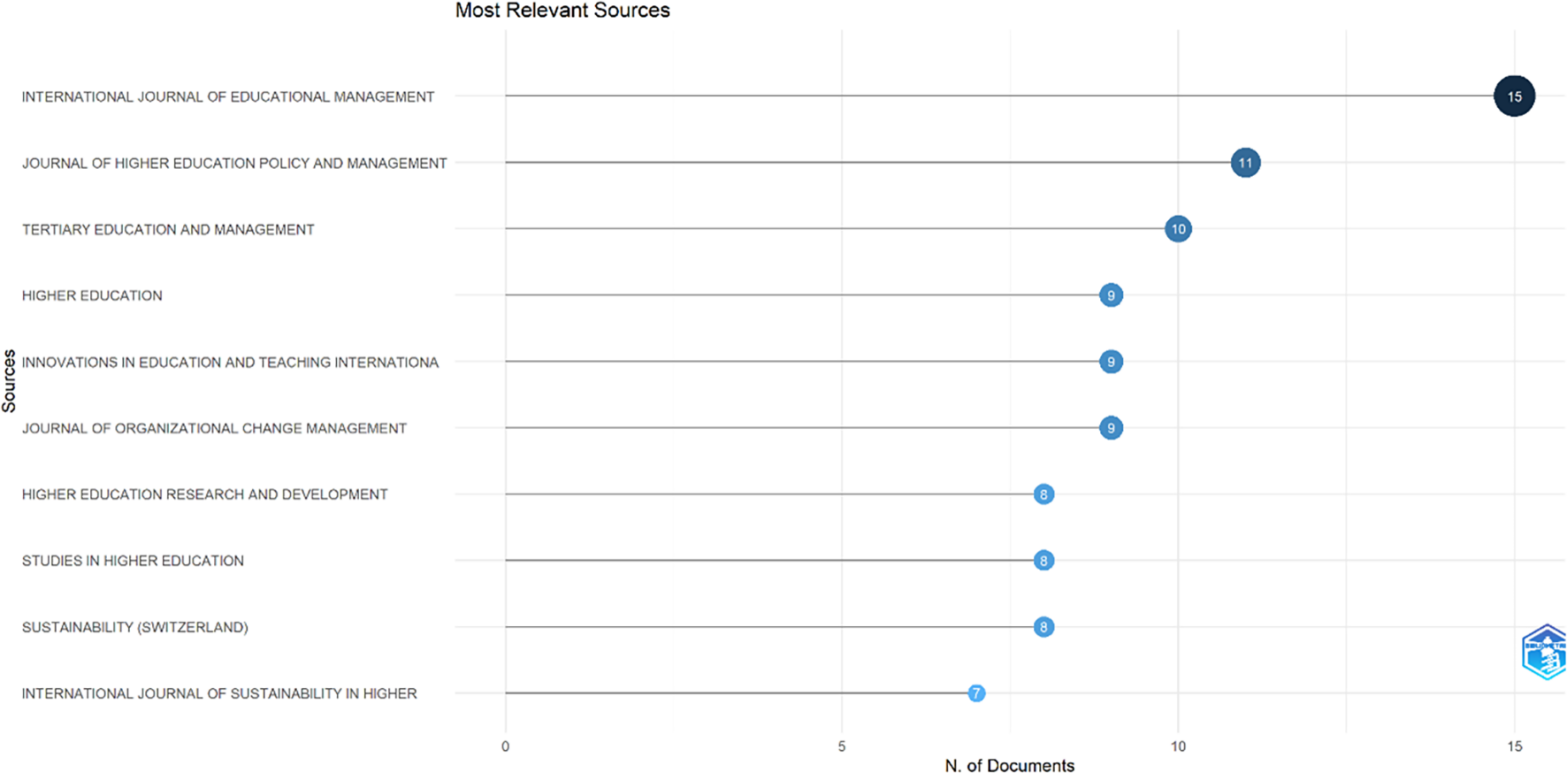
Most relevant sources.

This study searched for articles that discussed the relationship between collegiality, change management, and neoliberalism in higher education. Ten sources are the most relevant in Figure 5:

• 15 documents in International Journal of Educational Management
• 11 documents in Journal of Higher Education Policy and Management
• 10 documents in Tertiary Education and Management
• 9 documents in Higher Education
• 9 documents in Innovations in Education and Teaching International
• 9 documents in Journal of Organizational Change Management
• 8 documents in Higher Education Research and Development
• 8 documents in Studies in Higher Education
• 8 documents in Sustainability (Switzerland)
• 7 documents in International Journal of Sustainability in Higher Education

**Figure 6.**
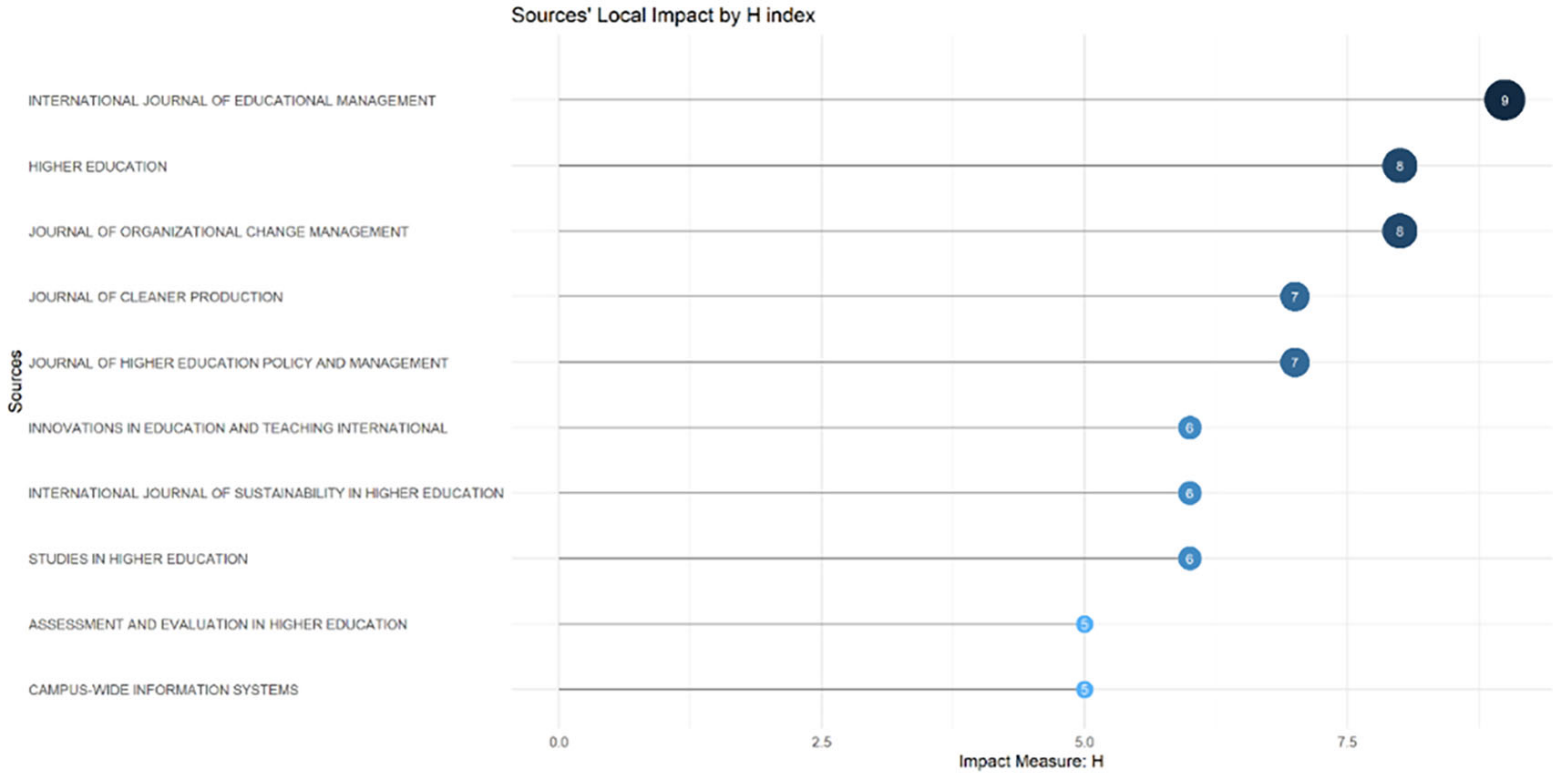
Sources’ local impact by H-Index.

**Figure 7.**
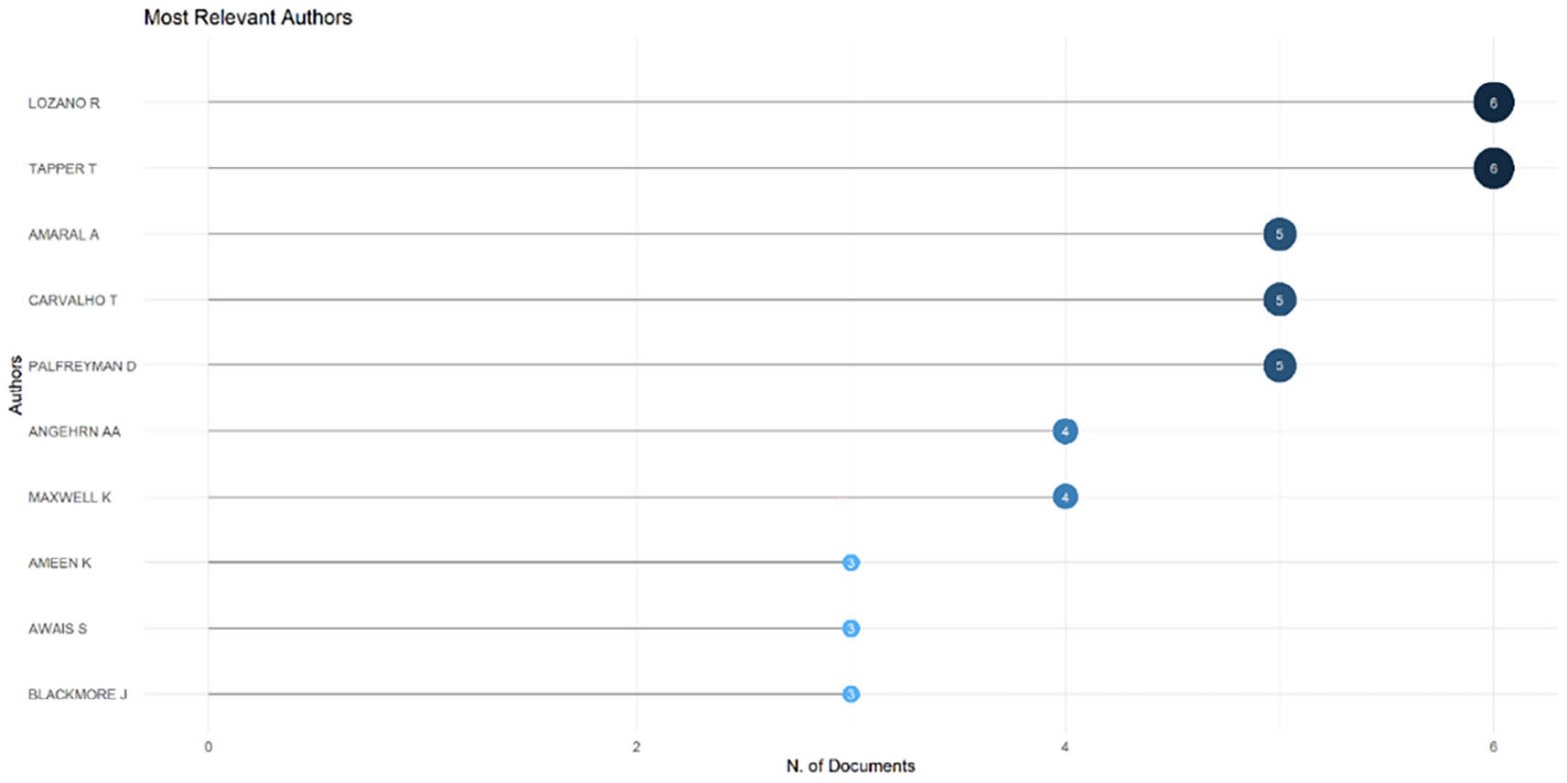
Most relevant authors.

**Figure 8.**
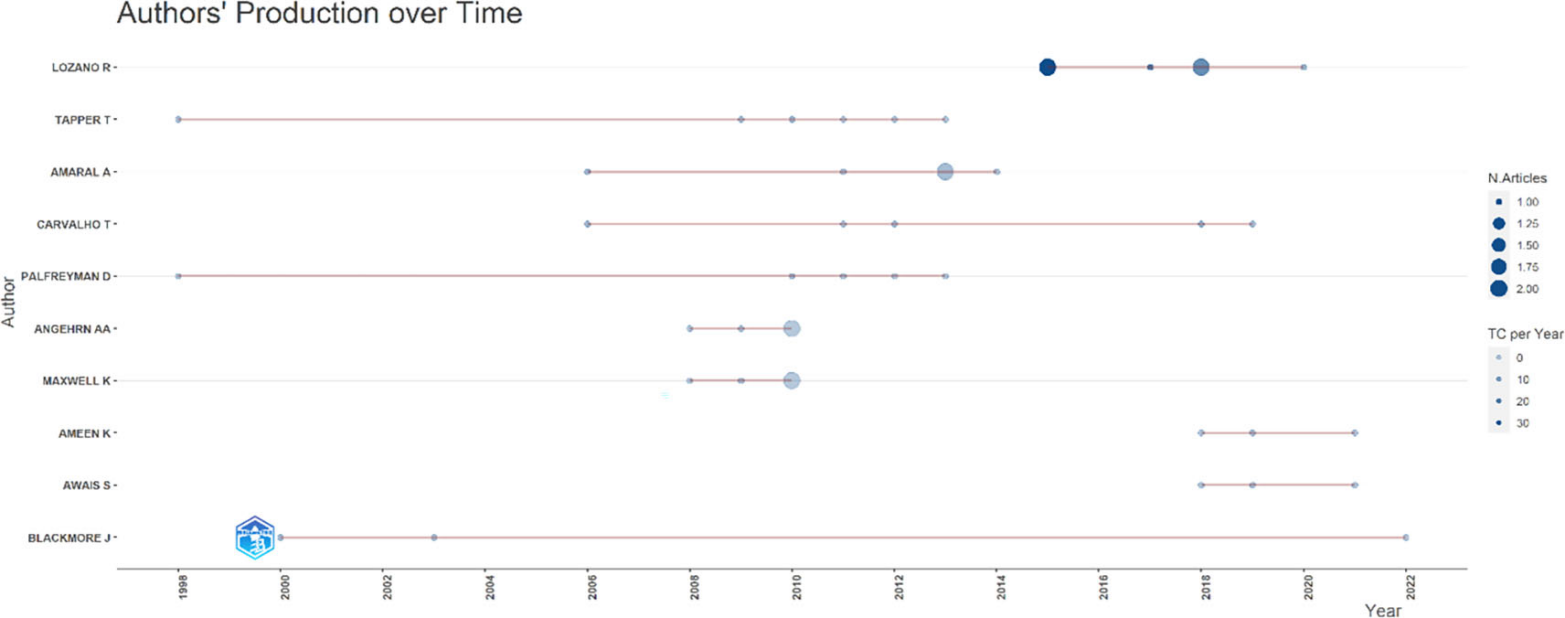
Authors’ production over time.

These sources are also supported by the sources’ local impact according to the H-index ( Figure 6), which shows that most relevant sources also have a high H-index. This indicates that journals in higher education dominate collegiality, change management, and neoliberalism. This could mean that those affected more by neoliberalism are nonprofit organizations in the education sector, especially in higher education. The researchers with the most relevance can be seen in Figure 7, namely those with articles published and indexed by Scopus until October 27, 2023. In Figure 7, it can be seen that researchers have relevance related to the relationship between collegiality, change management, and neoliberalism in higher education, which has the most significant relevance, namely Lazano R. and Tapper T. (6 documents), Amaral A., Carvalho T., and Palfreyman D. (5 documents), Angehrn AA and Maxwell K. (4 documents), Ameen K, Awais S., and Blackmore J. (three documents). Most relevant authors are also supported by authors’ production over time ( Figure 8), whereas researchers in most relevant authors are also authors’ production over time.

### Most relevant affiliations and countries

**Figure 9.**
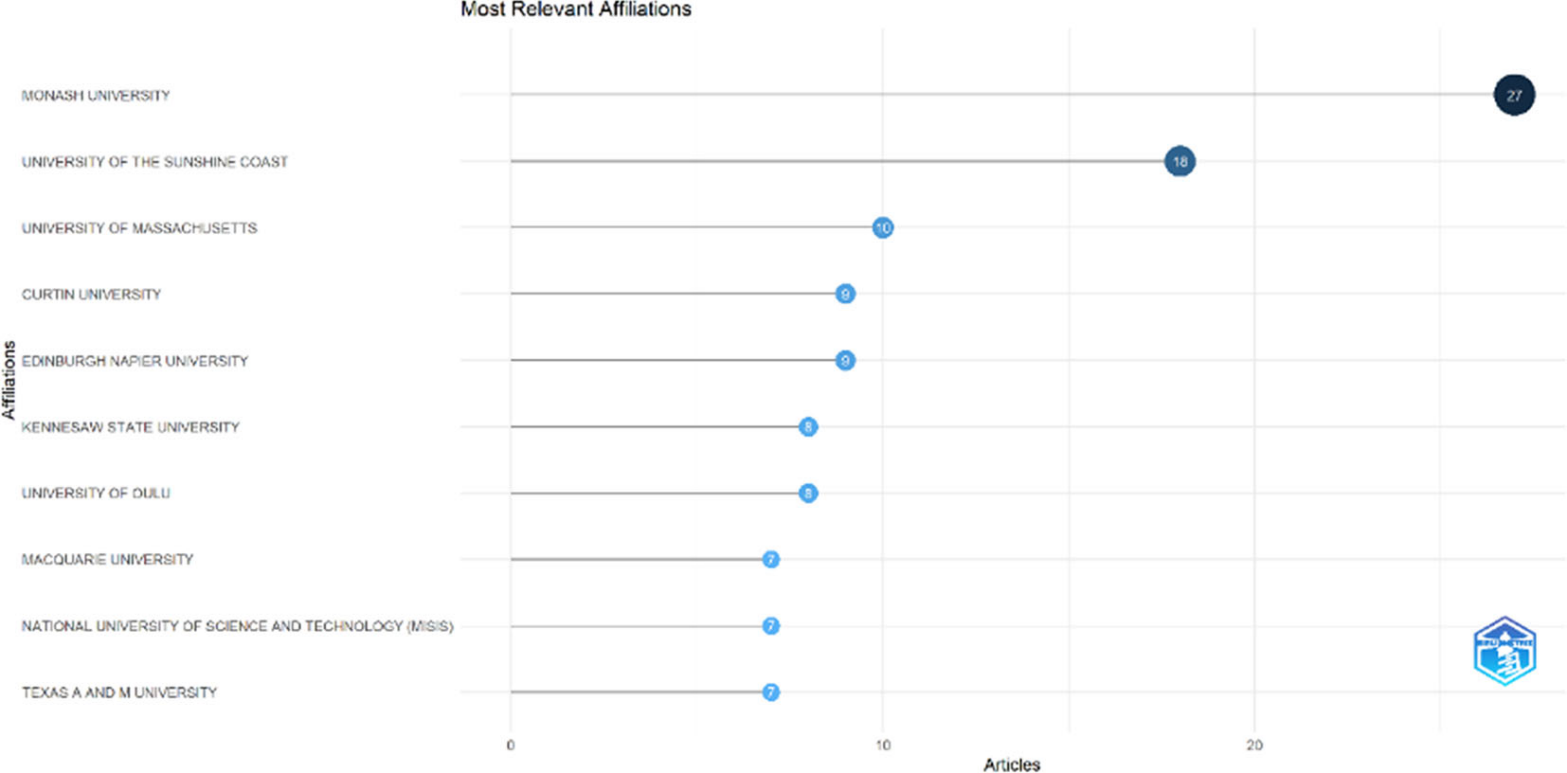
Most relevant affiliations.

**Figure 10.**
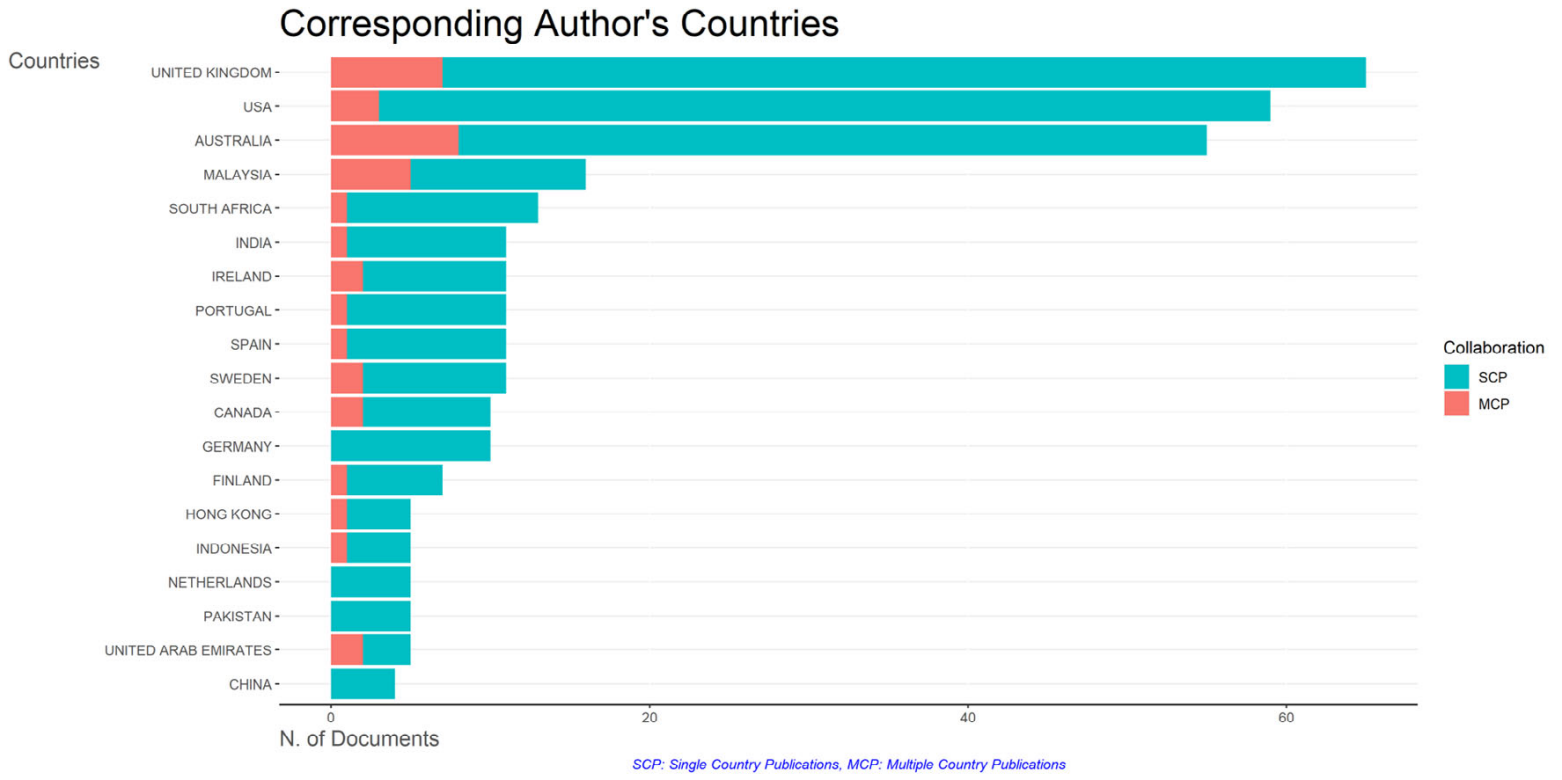
Corresponding author’s countries.

This section provides information about the affiliations that are most actively publishing scientific articles until 2023 related to the relationship among collegiality, change management, and neoliberalism in higher education according to Figure 9: Monash University (27 documents), University of The Sunshine Coast (18 documents), University of Massachusetts (10 documents), Curtin University (9 documents), Edinburgh Napier University (9 documents), Kennesaw State University (8 documents), University of Oulu (8 documents), Macquarie University (7 documents), National University of Science and Technology (Misis) (7 documents), and Texas A And M University (7 documents). The majority of affiliations related to relationships between collegiality, change management, and neoliberalism in higher education are still dominated by institutions from Australia and America while based on countries whose authors as correspondence are dominated by the United Kingdom, USA, Australia, Malaysia, South Africa, India, Ireland, Portugal, Spain, Sweden, Canada, Germany, Finland, Hong Kong, Indonesia, Netherlands, Pakistan, and the United Arab Emirates ( Figure 10).

The analysis of this author’s affiliation was intended to determine the affiliation that has resulted in many scientific publications on collegiality, change management, and neoliberalism in higher education. The results of this analysis can be used as a reference for future researchers to be used as a reference source for writing advanced articles and collaboration between authors, institutions, journal publication targets, and others.

### Co-occurrence network

**Figure 11.**
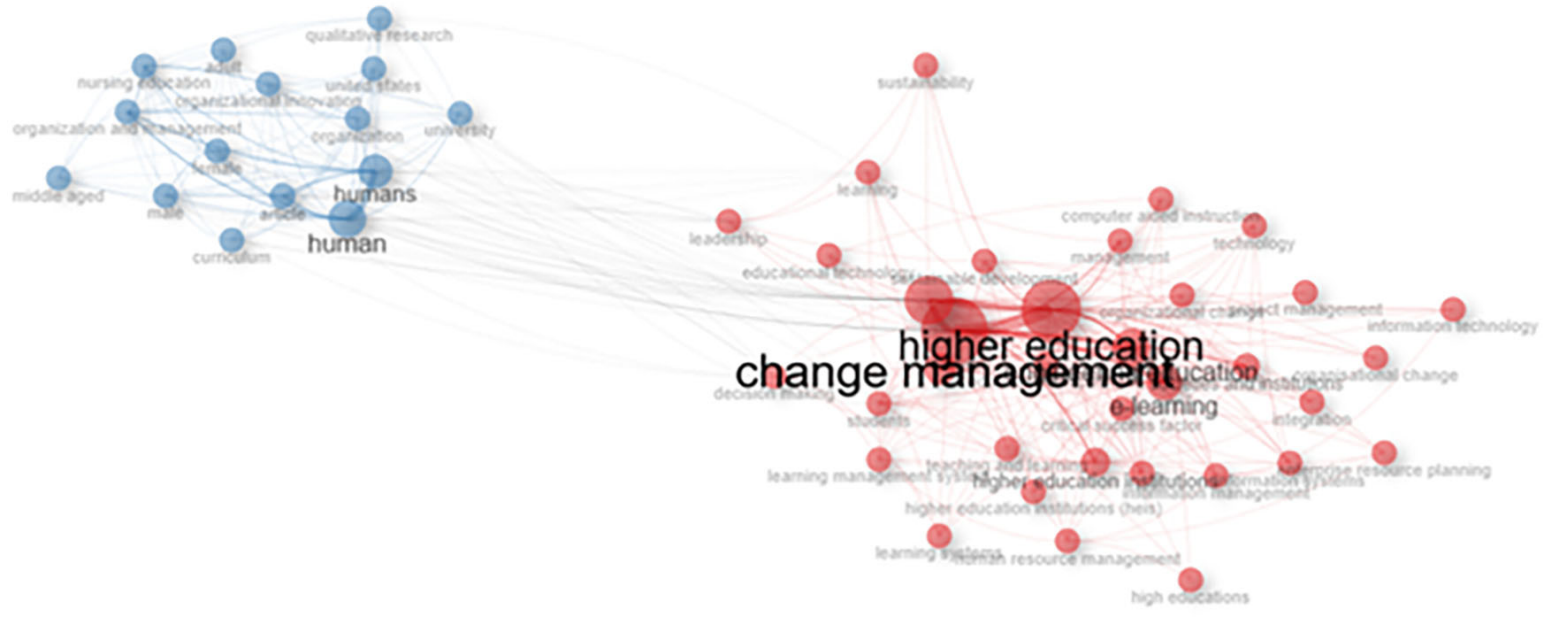
Co-occurrence Network.

Figure 11 shows a visualization of the keyword network, color, circle size, and font size. Meanwhile, the thickness of the connecting lines indicates the strength of the relationship between the keywords. The keywords in the image are displayed in the same color and are interconnected. For example, higher education and humans have larger font sizes than others, but have different colors. The line between higher education and change management in human beings shows a close relationship, and researchers have discussed much of the link between these two themes. Figure 10 shows that collegiality, change management, and neoliberalism in higher education are related to humans/humans, organizational innovation, learning, curriculum, organization, and management.

### Word Cloud and Treemap

**Figure 12.**
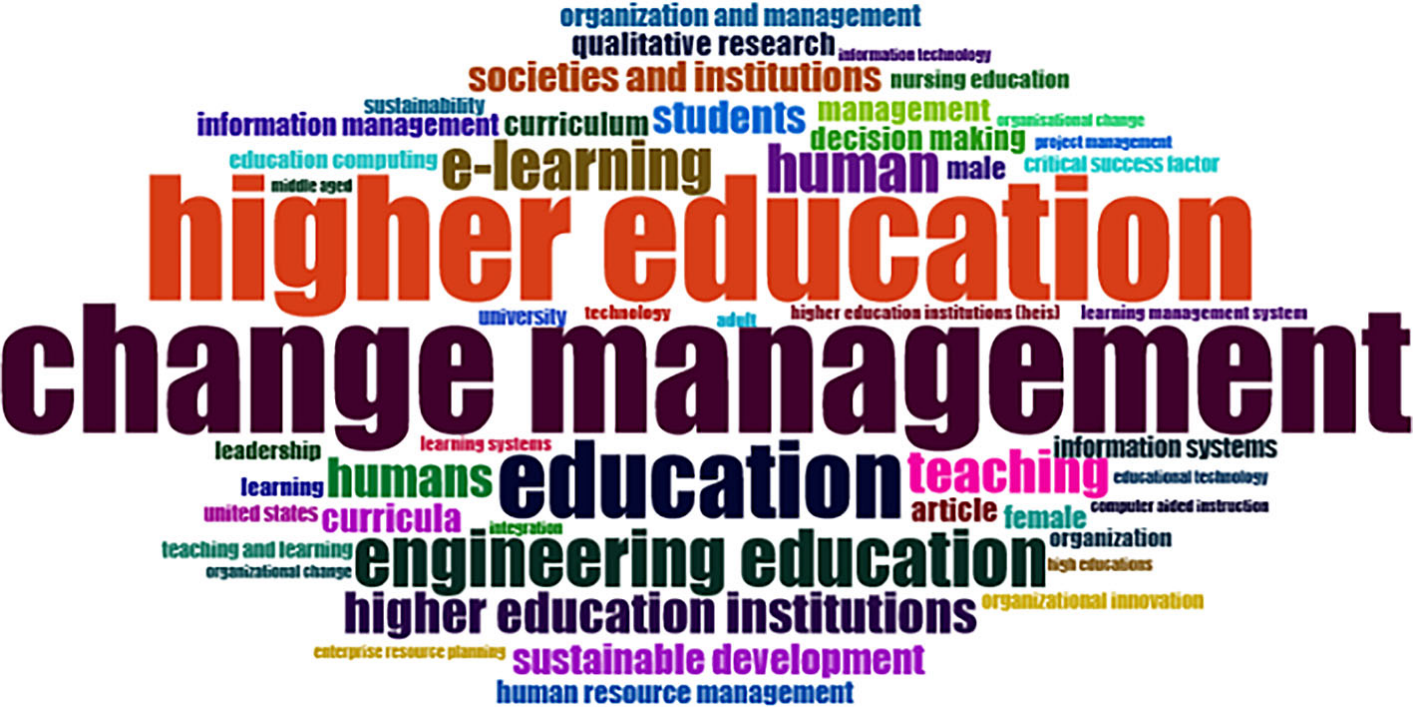
Word cloud.

**Figure 13.**
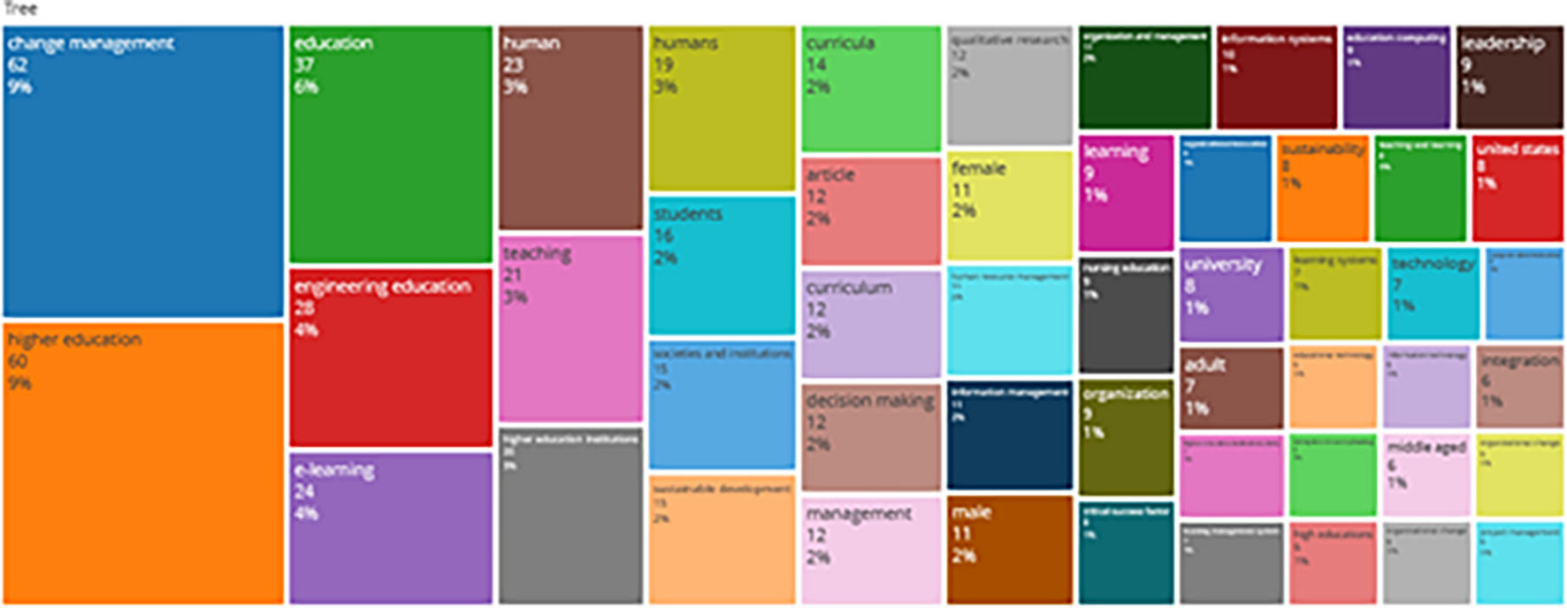
Tree map.

Figures 12 and 13 show the top 100 words based on the word cloud found in all articles on collegiality, change management, and neoliberalism in higher education, obtained as of October 2023. The size of each word represents the number of occurrences in the article’s title. The words shown in Figure 12 are title words trending toward collegiality, change management, and neoliberalism in higher education research that are integrated with other variables. The largest color indicates the most popular research title, whereas the same color indicates a connection. In Figure 12, the World Cloud appears irregular, but the dominant word in scientific publications is placed at the center to make it more visible with the maximum font.

Interconnected keywords are shown in the same color. For example, change management and education have color similarities of different sizes to show a close relationship. From Figure 13, it can also be seen that collegiality, change management, and neoliberalism in higher education are related to organization and management, leadership, curriculum, organizational innovation, organizational change, decision making, and humans. This can be interpreted as the relationship between collegiality, change management, and neoliberalism in higher education is related to leadership, organizational change, management, and humans.

This is supported by Croucher and Lacy (2022), which states that, in many changes that occur in higher education, a central role played by the holder of positions of authority and the culture of neoliberalism can be implemented through policies set by university leaders through budgeting mechanisms and internal organizational policies related to university governance. This opinion is in line with that of Osseo-Asare, Longbottom, and Murphy (2005), who conducted studies at Australian universities and stated that the ideology of neoliberalism has crossed the space of traditional liberalism. The institutional form of universities with a line management chain model that better maintains the philosophy of collegiality by maintaining a more collegial structure or democracy in a flatter structure has changed to a hierarchical model of authority that emphasizes more hierarchical market forces based on management dictated over the performance of work in the principal-agent chain of command. As the power of neoliberalism and corporate culture intensifies in universities, there are some things that can be seen as directly changing from the human side, such as changing the language used to present and evaluate human behavior and actions ( Giroux, 2002).

A shift in college management has occurred in many countries worldwide, with varying positive and negative outcomes ( Hamlin & Patel, 2017). Mercer (2009) and Ajayan and Balasubramanian (2020) in their research said that there are four things related to the ideology of neoliberalism in the form of *managerialism* in universities: 1) increasing internal and external accountability of organizations, where universities have to control the quality of research and teaching based on output, especially graduation rates, publications, and performance indicators; 2) related to the marketization of universities, where universities are becoming more customer-oriented and stimulated academic competition for students, such as getting published in reputable journals; 3) increased emphasis on efficiency where there are restrictions on government and universities funding are required to be able to provide maximum services by reducing service costs; and 4) managerialism promotes entrepreneurial activities by building solid relationships with various stakeholders, collaborating with industry, and finding various alternative short-term and long-term revenue mechanisms to generate revenue. However, this may come at the expense of academic freedom and collegiality ( Nickson, 2014). Following some of these assumptions, we can understand that implementing neoliberal ideology in the form of NPM/ *managerialism* has shifted the identity of higher education collegiality towards governance that prioritizes corporatization management.

An exciting thing that researchers want to criticize here after seeing the results of the bibliometric analysis is the idea of thinking of world scholars. If universities prioritize market forces, what about collegiality in higher education? The theoretical foundation of corporate culture and its role in corporate governance are principally derived from agency theory ( Ballantine, Berle, & Means, 1932; Christopher, 2012b). This theory is certainly more in favor of the interests of principals (shareholders), while university management is certainly very different. Therefore, the impact of changes resulting from the implementation of neoliberalism on collegiality in universities must be balanced, lest the values that universities should carry in creating community welfare through the development of science and social justice for the entire community is eroded by the corrosive effect created by market culture that prioritizes business agendas in universities ( Croucher & Lacy, 2022; Gaus & Tang, 2023). Do not allow the common goal of establishing universities to create community welfare further away from the original goal. Therefore, each university must realize that this change is inevitable, but must be able to immediately adapt to all the consequences of adapting to the new environment created by the ideology of neoliberalism, which presents some significant challenges for change and increasing the competitiveness of universities.

## Discussion

The forces of globalization have driven the literature review. Changes in higher education have several dimensions. One of the dimensions of globalization is neoliberalism, which implements market forces in higher education. This paradigm shift presents several consequences in higher education, not only on the education side but also on the governance of universities. The results of the bibliometric analysis of the articles used in this study presented a clear and broad view. In this article, we also want to provide a view in accordance with what the researcher captured from a review of research results from previous researchers with a tendency to focus on research in the last five years. Although not in their entirety, the selected articles are featured in this review.

As discussed earlier, the implementation of the ideology of neoliberalism has been widely studied and has attracted the attention of several researchers ( Fleming, 2020; Gaus & Tang, 2023; Gaus et al., 2019). The term neoliberalism is more familiar with the terms corporatization, managerialism/new public management (NPM), and accountability, which explicitly reduce government interference and replace it with the market, assuming that the market is considered the most effective allocator of resources, can encourage innovation and the ability to be entrepreneurial. Universities affected by neoliberalism tend to become more *market-oriented *, considering technology/digitalization in universities, such as the emergence of many virtual universities, and it is increasingly important for universities to collaborate with strategic alliances and partners to form networking for universities ( Dopson et al., 2019).

Neoliberalism in higher education has become an increasingly significant topic in academic discourse, particularly concerning its impact on collegiality and academic freedom. Research conducted in various countries, including Sweden and Australia, indicates that the growing market orientation within universities has altered academic structures and cultures. For instance, Macfarlane (2019) illustrate how academic norms have shifted in an era of high performativity, where academics feel compelled to compete and produce measurable outputs.

In Australia, government-led higher education reforms, as described by Bay (2011), have created an environment where universities operate more as corporate entities than as public institutions. These reforms emphasize the importance of accountability and performance measurement, which, in turn, diminishes the space for collegiality and free academic discourse. This trend is also evident in the study by Olssen and Peters (2005), which demonstrates that neoliberalism has transformed higher education into an arena of intense competition, where knowledge is treated as a commodity.

Our findings also reflect previous research in Southeast Asia, where the influence of neoliberalism is evident in changes to university governance. Hwami (2013) indicates that in Zimbabwe, neoliberal policies have led to the privatization and commercialization of higher education, creating inequities and enriching elites. This suggests that, despite differing local contexts, similar patterns can be observed across various countries, including Southeast Asia, where universities are also adapting to more corporatized governance models.

By expanding this context, we can better understand how similar or divergent findings emerge in both local and global contexts. Phillips (2017) emphasizes that the presence of neoliberalism in Australian higher education has transformed the university research environment, impacting the employability of graduate students. This indicates that the effects of neoliberalism are not confined to educational policies but also permeate daily practices within universities, influencing how academics and students interact and collaborate.

This study summarizes some of the changes in higher education as an influence of adopting neoliberalism, addressing eight central issues related to autonomy, quality assurance (QA), internationalization, market orientation, managerialism, entrepreneurialism, digitalization, collaboration, and leadership. Researchers expect these eight issues to contribute to leaders in higher education making strategic decisions to institute changes in their universities. As input material on the pattern of changes in the orientation of higher education governance, we also complete this research from several points of view, including the differences between educational and business organizations (see Table 2).

**Table 2.** Differences between business organizations and educational organizations.

Viewpoint	Business organization	Educational organization
Policy	Market-oriented	Speak knowledge
Organizational focus	Efficiency of operations through achieving maximum profit at minimum cost for survival and growth in the face of competition	Provide professional services where well-being is considered to be the primary concern of the organization’s services
Institutional role	A business is a provider/producer	Universities are service providers, regulators, and standards makers
Control	In a heteronomous organization, the presence and absence of resources controlled by managers	Professional workers carry out autonomous organizations and the presence and absence of control over resources.
Decentralization	Decentralized to improve adaptability to flexible markets ( Kreysing, 2002)	A decentralized organizational structure might bring strong particularistic interest
Leadership	• Traditional. Business leadership is based on the concept that leaders are central to task performance and maintenance of human resource output, emphasizing individual performance equality • Contemporary. Business leadership is to promote adaptive change by developing a vision of the future and a change strategy	• Traditional. Academic leadership is characterized by personal academic achievements such as publications in accredited journals, international conferences, and responsibility for academic development • Contemporary. Academic leadership focuses on leadership qualities, effective and efficient administrative mechanisms, and nuances of interpersonal relationships and empathy
Leader role	The role of the leader is to overcome crisis (financial)	The role of the leader as an ideology of heroism is not only in times of crisis
Prosecutor central	The Central Actor is the manager	The central actors are the central leadership and faculty
Decision	Hierarchical levels in the organizational structure are used as the basis for decision-making	In collegial relationships, decision-making is seen as a process; competence is more important than ranking in organizations
Central issues	Customer satisfaction is a central issue	Intellectual freedom is at the core of the goal of truth-seeking and knowledge creation
Customer	Customers are at the core of quality	Graduate and Student Users
Quality indicators	Customer Satisfaction is a key indicator of quality	More than satisfying customers, but to shape the behavior of stakeholders
Administrator roles	Administrator roles protect the interests of institutions and customers	Education completion services
Academic	Academics are viewed as employees by institutions, as members of the communities they support, and also as contributors to ministry causes	Academics have freedom, which is veto power over every decision made by the institution
Performance	Relying on remuneration	Rely on moral engagement and expressive performance
Performance measurement	Performance measurement is a straightforward and considerable technical procedure (customer satisfaction)	Performance evaluation is complex and not only dominated by customer satisfaction
Accountability *vs.* moral conviction	As an important fact, managers carry out an ethics of accountability or consider the possible consequences pragmatically	Academics are grounded in core ethical values and moral convictions
Price *vs.* value	Price is everything	Value is everything
Knowledge	Knowledge is a commodity	Knowledge is adventure
Output	Output can be easily measured	Output is much more difficult to measure, and measurements are rarely made

( Ajayan & Balasubramanian, 2020; Fahmi, 2009; Fugazzotto, 2012; Prysor & Henley, 2018).

**Figure 14.**
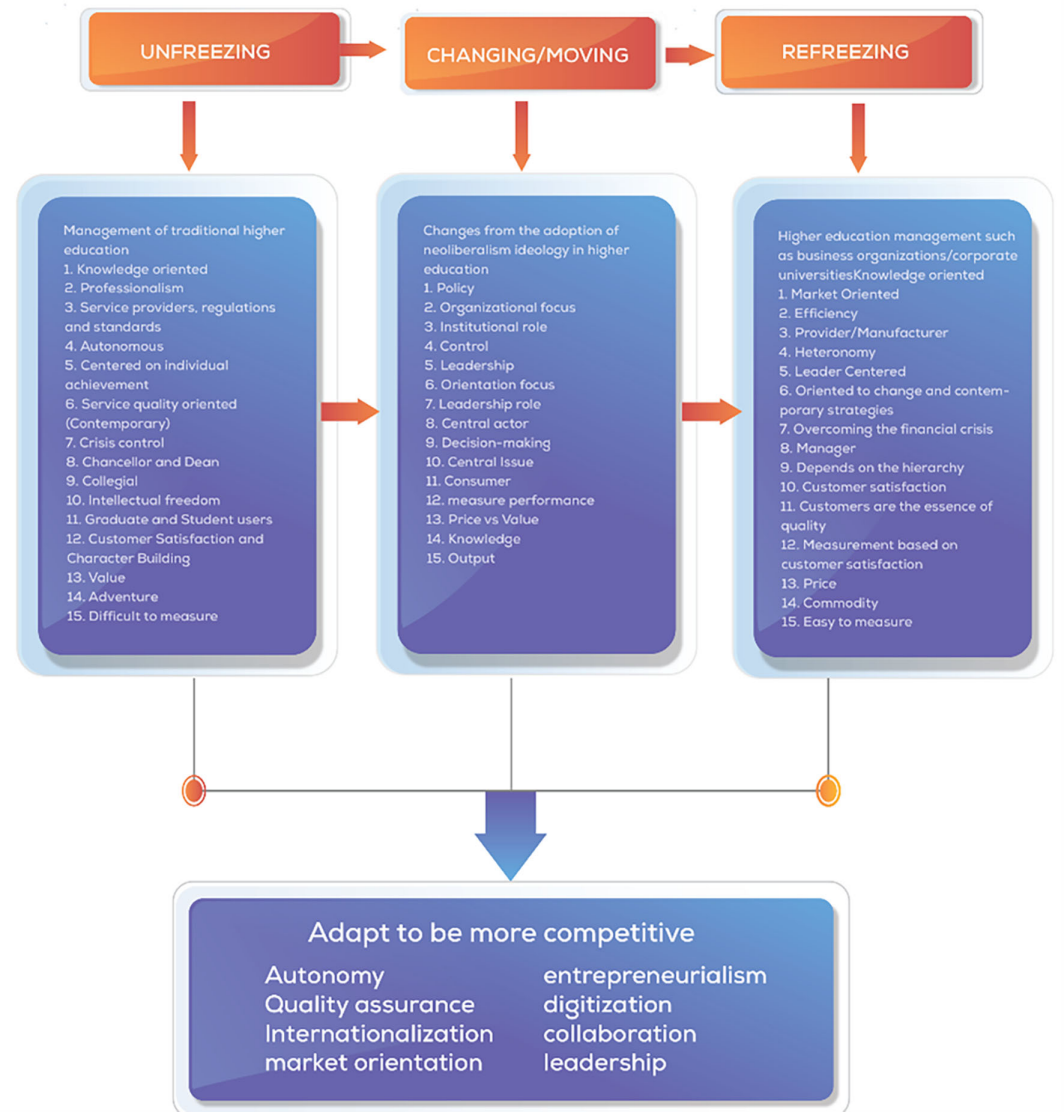
Comprehensive change patterns.

The acceleration of change in each university will differ depending on how much the organization is ready to respond to challenges and the urgency to adapt to change quickly. The transformation process of change comprehensively considers changes in higher education. We present a framework of thought adapted to Lewin’s (change management and aspects related to various perspectives of change to face the environment of neoliberalism in higher education. The Lewis Change model was used in this study to identify changes occurring in educational organizations and the adoption of what should be re-institutionalized from a change process ( Figure 14).

### Autonomy (Self-government)

Some countries have focused on developing the concept of autonomy as part of transforming higher education governance into autonomy or self-governance. This is a result of the emergence of the concept of New Public Management (NPM), which began to shift government control into market power (( Nasution, Prasojo, Jannah, & Yumitro, 2020). Influencing forces include various stakeholders and the dynamics of the relationships (agreements) between stakeholders and university governance. Christopher (2012b) details five factors that have influenced the paradigm of higher education governance: 1) Central Government, 2) Local Government, 3) global competition, 4) collegial governance, and 5) internal management.

Granting autonomy to universities is expected to bring about broad changes to the governance and management of institutions. Autonomy refers to “the power and authority of an institution to run its affairs without undue influence or direction from the government” ( Taib & Abdullah, 2015). Autonomy can be divided into two types: 1) substantive autonomy and 2) procedural autonomy. Substantive autonomy includes academic and research fields, particularly autonomy related to curriculum design, research policy, degree awarding, and so on. In comparison, procedural autonomy covers non-academic fields that have much to do with financial issues such as borrowing funds, spending budgets, and managing salaries.

Granting autonomy to public universities entails bestowing autonomy in four domains: The first aspect is organizational or institutional autonomy, which entails bestowing universities with the power and authority to establish policies, devise procedures, and make choices to fulfill higher education objectives and purposes through effective governance and accountability frameworks. Financial autonomy refers to the granting of power and authority to Public Universities to create policies, establish procedures, and make decisions regarding financial and revenue management. This is accompanied by a requirement for accountability, transparency, integration, and implementation of effective and efficient management practices ( Abdullah & Osman, 2015). Human resource autonomy refers to the level of authority granted to Public Universities to formulate policies and establish processes for organizational development, remuneration, and employee development. This autonomy enables universities to attract, nurture, and retain highly skilled professionals and talented individuals. Furthermore, autonomy in the academic sphere entails endowing state universities with the power and authority to formulate policies in areas such as the establishment and discontinuation of study programs as well as the advancement, execution, and assurance of academic excellence, research and development, innovation, and knowledge dissemination ( Taib & Abdullah, 2015). Therefore, granting autonomy helps universities improve their quality through quality assurance.

### Quality assurance (QA)

The second primary concern revolves around quality assurance in higher education, which refers to the public’s duty to exhibit commendable levels of performance. This matter has garnered significant attention and sparked extensive debate, making it a crucial strategic issue in education systems worldwide in recent years ( Fahmi, 2009). Ensuring quality and obtaining certification are essential responsibilities and priorities for institutions. Universities can accomplish their objectives of meeting the requirements of students, communities, and stakeholders regarding high-quality education, training, and research by engaging in quality assurance activities, thereby enhancing their standing. Quality assurance standards and accreditation are the most effective ways to assess the degree of quality of educational services. Quality assurance aids in identifying issues and constraints that need to be rectified and resolved before implementation. Implementing quality assurance and accreditation is a crucial factor contributing to the success of most higher education institutions globally ( Seyfried & Pohlenz, 2018).

Quality assurance is a contemporary principle within the Total Quality Management (TQM) framework. This serves as a means for firms to identify strategies to enhance and advance overall workforce performance. Total quality (TQ) is a component of Total Quality Management (TQM) in education. The TQ, or Total Quality, is a collection of standards and procedures for enhancing educational products. It encompasses technical requirements, the desired attributes of educational products and processes, and the necessary procedures to meet these specifications. Quality assurance encompasses all activities for evaluating and enhancing the value of one or more standards during implementation. The organization guarantees that the products or services adhere to the established quality standards. Total Quality (TQ) is an essential component of quality management that aims to assure individuals and communities by ensuring that quality criteria are met ( Hanh, 2020).

The contemporary notion of Total Quality Management (TQM) in educational institutions has demonstrated that quality assurance in higher education is an ongoing and systematic procedure for evaluating quality based on predetermined criteria and standards. Hanh (2020) mentioned several indicators of higher education quality assurance, including

1. *Strategic management:* This metric assesses a university’s development and implementation of its long-term plans and objectives. Strategic leaders are responsible for assessing the current state of higher education institutions, evaluating their strengths and weaknesses, and developing strategic plans that encompass the organization’s vision, mission, and educational objectives. These plans guide an institution’s short- and long-term actions.
2. *Quality management methods:* This metric evaluates the capacity of educational institutions to deliver services that align with the expectations of society, employees, students, job market, and other stakeholders.
3. The marketing and customer service indicators identify societal needs, labor market trends, and learners to offer successful and suitable training and educational services.
4. Human resource development: This metric encompasses the ongoing training of personnel to enhance their ability to efficiently perform tasks and achieve optimal productivity.
5. The equal opportunity indicator ensures that educational institutions and the job market provide equal opportunity. This leads to higher employee and unit satisfaction, and improves labor productivity and quality.
6. *Health and safety:* Maintaining a healthy and safe environment for employees, students, and beneficiaries is a crucial measure of an educational institution’s performance.
7. *Contact management:* This metric requires educational institutions to fulfill the requirements of both students and employees and effectively disseminate information across all levels of the organization.
8. *Consulting services:* This indicator pertains to activities related to organizational governance that aim to identify the requirements of diverse learners (including psychological, academic, and social needs) and devise strategies to fulfill them.
9. *Program design and implementation:* Learning programs should be developed according to the requirements of the job market and student demands.
10. *Certificate of rank:* This indicator verifies that students receive qualifications that align with their capabilities.

By incorporating the above criteria and indicators, developing a set of key performance indicators is a systematic approach for ensuring quality in higher education. The ultimate objective is to produce superior outcomes that align with societal and labor market demands while also ensuring customer satisfaction in the long run. Colleges frequently establish their reputations by obtaining accreditation certificates that validate their commitment to ensuring the quality of their graduates when they enter the job market. Various educational theorists have proposed numerous views of accreditation. However, they unanimously concur that accreditation is a systematic process within an organization that seeks to advance and enhance educational institutions and their programs ( Seyfried & Pohlenz, 2018). Accreditation is an indicator of the quality of education management in higher education as a whole, and international ranking agencies require international accreditation to measure higher education performance. Therefore, a change leader must realize the importance of maintaining the quality of education to increase competitiveness at his university.

### Internationalization

Promoting internationalization as a crucial policy for education reform can enhance the potential and competitive advantage of higher education institutes to compete nationally and internationally. Internationalization is frequently incorporated as a strategic approach in colleges, representing a robust endeavor by university administrators to enhance their standing in the global market. The primary determinant of this pattern is the worldwide assessment of educational establishments facing global rivalry ( Engwall, 2016). Internationalization is an ongoing effort with an evolutionary quality or the development of concepts ( Knight, 2008).

By the mid-1990s, Knight (1994) introduced an organizational process or approach to internationalization at the institutional level had been introduced by Knight (1994), defining internationalization as “the process of integrating international and intercultural dimensions into teaching, research, and institutional service functions.” This definition has been widely used to describe internationalization ( Guo & Guo, 2017; Ota, 2018). However, this definition is limited to the institutional dimension. Knight (2008) proposes a new definition, which states that internationalization at the national, sector, and institutional levels integrates intercultural or global dimensions into the purposeful functioning of higher education. In addition, Hudzik (2014) provides a more comprehensive definition of internationalization, defining it as deliberate institutional commitments and actions to embed and integrate international, global, and comparative content and perspectives across higher education teaching, research, and service missions. Beyond the basic functions of such institutions, Hudzik argues that a comprehensive approach is the desire to integrate internationalization into the ethos, values, and institutional mission of higher education ( Hudzik, 2014). Furthermore, Hudzik emphasized that for “comprehensive Internationalization, it is important to understand by leaders of universities, government, faculty, students, and all academics, as well as other support service units ( Hudzik, 2011).

According to Knight and Hudzik’s definition, internationalization is a complex and diverse process that incorporates international, intercultural, and global elements into the functions and goals of higher education institutions. Its purpose is to enhance the quality of education and research for all students and staff, while making significant contributions to society ( Guo & Guo, 2017). Therefore, the internationalization program must be designed to be compatible with the internal structure or existing educational and research activities to improve the international image of the university externally without fundamentally changing its substance, because internationalization is not a stand-alone goal or objective, but aims at the reform of universities for quality improvement from a global point of view through internationalization is the way and process to realize competitive advantage during global competition ( Ota, 2018). A leader of change who wants to compete on an international scale cannot ignore the internationalization factor.

### Market orientation

Rivals are crucial in determining a market’s focus and approach ( Kohli & Jaworski, 1990; O’Dwyer & Gilmore, 2017; Sukoco et al., 2021). Organizations with the capacity to discern opportunities and dangers from external entities can predict development and establish strategies to adjust to change ( Slater, Mohr, & Sengupta, 2014). It is imperative to embrace a market orientation within an organization as the initial step towards a transformative process ( Lafferty & Tomas M. Hult, 2001; Narver, Slater, & Tietje, 1998; Sukoco et al., 2021). Klarner et al. (2007) contend that companies should possess the capacity to be highly responsive in order to adjust to changes, particularly in the market effectively. Adaptability can be assessed by a heightened capacity to obtain information regarding present and forthcoming market circumstances ( Najafi-Tavani, Sharifi, & Najafi-Tavani, 2016). Market demand fluctuations prompt enterprises to prioritize data acquisition for evolving customer demands, interests, and behavior. This enables them to deliver exceptional customer value by leveraging diverse information sources ( Narver & Slater, 1990). Engaging in activities to acquire and analyze market information will facilitate the cultivation of a culture of learning inside firms ( Deshpandé, Farley, & Webster, 1993; Deshpande & Webster, 1989).

Leaders who embrace market orientation actively solicit prompt consumer input and provide unrestricted access to extensive information for all organization members ( Jaworski & Kohli, 1993; Pinho, Rodrigues, & Dibb, 2014). The unrestricted availability of information within the organization enables personnel to acquire and embrace new knowledge about the market, thereby improving and refreshing their skills and understanding throughout the organization. This empowers them to consistently make proper decisions and take necessary action ( Minucci, 2016). This subsequently generates additional value for customers ( Liu et al., 2002) and optimizes the service delivery process more effectively and efficiently ( Siggelkow & Terwiesch, 2019).

Moreover, leaders in companies must devise methods to establish a collective understanding ( Gebhardt, Carpenter, & Sherry, 2006) and enhance cohesion to establish a mechanism for collective transformation ( Liu & Atuahene-Gima, 2018). Market orientation in non-profit organizations aligns the organization’s attention towards external factors such as stakeholders, thereby consolidating the organization’s emphasis on crucial external elements ( Modi, 2012) and internal constituencies. In addition, universities involve numerous stakeholders who can shape organizational policies ( Mainardes, Alves, & Raposo, 2012). The institution prioritizes customer orientation towards students and the community, who are recognized as stakeholders. Students immediately utilize and benefit from the products and services offered by the university ( Akonkwa, 2009); higher education is widely seen by society as an institution that safeguards and perpetuates sources of knowledge ( Lu & Etzkowitz, 2008), the organization is anticipated to play an active part in the process of transforming knowledge into commercial products and services within the innovation value chain ( Urbano & Guerrero, 2013).

Consequently, institutions should be attentive and adaptable to the desires of students and society, offering the options that they seek ( Nixon, Scullion, & Molesworth, 2010). Colleges prioritizing customer satisfaction can oversee service operations, distribute essential information to all stakeholders, and actively strive to meet their demands ( Hammond, Webster, & Harmon, 2006). This method establishes an environment favorable for colleges that prioritizes market-oriented approaches ( Nagy & Berács, 2012). Universities that possess the ability to offer genuine and groundbreaking services while being cognizant of the requirements of their consumers, including students and the community, can collaboratively design new service formats and foster a culture that prioritizes customer satisfaction ( Dollinger & Lodge, 2020; Dollinger & Vanderlelie, 2021; Sukoco et al., 2021).

Organizations that effectively adapt to the accelerating pace of change must consider customer preferences and competitive actions ( De Luca & Atuahene-Gima, 2007). To achieve a comprehensive implementation of change and strategy across the entire organization, it is necessary to disseminate them sequentially ( Klarner et al., 2007). Organizations with robust interfunctional coordination exhibit efficient communication and collaboration among several departments ( Marsh & Stock, 2003). To foster a unified culture that maximizes the use of market intelligence for institutional progress ( Gebhardt et al., 2006). Leaders in higher education who can capitalize on opportunities in the commercial aspects of higher education are expected to produce revenue from non-academic activities. The perception of higher education as an institution has shifted, recognizing it as a crucial catalyst for economic growth and development ( Taib & Abdullah, 2015).

### Entrepreneurialism

Since the outset, it has been universally acknowledged that universities’ primary function is to serve as a facilitative instrument to foster economic growth and development. Nevertheless, modern colleges are embracing a more business-oriented approach to foster and facilitate economic growth. With the emergence of a new paradigm, universities have transformed into entrepreneurial institutions, actively engaging in the commercialization of research and adopting a proactive stance towards the role of academic research in the marketplace. This paradigm emphasizes that the environmental forces of a hypercompetitive global environment have transformed the university’s mission of research, teaching, and service. The increasing number of “business-like” higher education institutions worldwide puts pressure on universities to become more entrepreneurial and achieve higher efficiency in improving quality ( Ajayan & Balasubramanian, 2020). Recent developments in financial autonomy granted by the government to several universities for financial management flexibility, especially to state universities, have several consequences, such as reducing government subsidy funds, which means that autonomous universities must generate some alternative income independently ( Amran & Muhammad, 2014). This is in line with managerial, which requires universities to be more entrepreneurial and find alternative income mechanisms in universities ( Ajayan & Balasubramanian, 2020).

Some researchers have determined that universities can adapt to and become entrepreneurial universities ( Clark, 1998; Sporn, 2001). Clark (2000) argued that many universities should become more proactive and entrepreneurial. The primary emphasis of the university’s entrepreneurship center lies in exploring innovative approaches to earn revenue and is seamlessly incorporated into the strategic planning framework of collaborating institutions. The primary responsibility of the entrepreneurship center is to execute strategic initiatives about fundraising and engage external specialists to transform concepts, typically originating from students and research endeavors, into commercially viable goods. Hence, entrepreneurship has the potential to stimulate trade and business collaboration and facilitate the provision of research and consulting services from partner universities to both public and private entities ( Fahmi, 2009).

Another assumption that universities are increasingly entrepreneurial is the consequence of granting autonomy to the government, which results in a reduction in public funds by the government by removing subsidies and giving total confidence that universities have been able to be independent regardless of government control ( Gaus, 2019a). Therefore, universities cannot only rely on income from the primary sector, namely academic income, but must also develop a pattern of partnership/collaboration to obtain funding from the non-academic business sector. The ability of a change leader to optimize all additional business potential from non-academic pathways greatly determines the sustainability of the university he leads. Each university’s adaptability in adjusting to technological developments/digitalization in education provision cannot be ruled out.

### Digitalization

Digitalization concerns using technology to renew, simplify, and increase processes, tasks, and products ( Tomte, Fossland, Aamodt, & Degn, 2019). In the last decade, all aspects of life and business operations have been significantly transformed by digital technology. An organization’s digital transformation principally concerns the adoption of technology portfolios such as the Internet of Things (IoT), digital platforms, social media, Artificial Intelligence (AI), Big Data, and Machine Learning (ML) ( Antonopoulou, Halkiopoulos, Barlou, & Beligiannis, 2021; Foerster-Metz, Marquardt, Golowko, Kompalla, & Hell, 2018; Khalid et al., 2018; Matalamäki & Joensuu-Salo, 2021). On a broad scale, the shift to modern technology has brought about new competitive mechanisms, structures, work systems, and interactions. On a smaller scale, digitalization impacts the dynamics, processes, skills, and competencies required by all members of the organization, regardless of their position ( Cascio & Montealegre, 2016; Rienties, Brouwer, & Lygo-Baker, 2013).

The process of digitalization in education encompasses a wide range of factors that contribute to its quality, including organizational matters, technology infrastructure, and pedagogical methods ( Cascio & Montealegre, 2016; Selwyn, 2011), and it influences internationalization by offering more flexible online education programs ( Conole, 2014). In addition, digitalization provides conveniences such as administrative solutions, data security systems, systems to detect fraud and plagiarism, research data storage, library services, and various learning resources as well as opportunities for better collaboration across campuses ( Khalid et al., 2018).

Digitalization has significantly changed higher education due to technological developments and globalization. Universities’ ability to rapidly increase digitalization will affect their ability to survive global competition, especially in internationalization, student mobility, funding, and world ranking positions ( Khalid et al., 2018). This is in line with previous research ( Wu, Yeniyurt, Kim, & Cavusgil, 2006) showing that Information Technology (IT) can contribute to network collaboration and strengthen an organization’s competitive capabilities. College leaders must recognize the need to evolve and reshape structures, processes, pedagogic practices, curricula, and innovation. Meanwhile, there is no innovation without collaboration. This is in line with Luthans (1988), who states that the success of a leader depends on his ability to build networking.

### Networking and collaboration

The resulting innovation performance excellence in collaboration depends on the ability to manage *networking* ( Ter Wal et al., 2020). Networking is a set of relationships connecting people inside and outside an organization that can be relied upon to succeed in work ( Altbach, 2011). Collaboration with strategic partners is urgently required to address the challenges of neoliberalism. Social capital theory states that networks are a power source for individuals and organizations ( Timberlake, 2012). Networking can potentially increase career success for individuals, such as raises, promotions, career satisfaction, employment opportunities, job performance, social support, resources, and professional support ( Seibert, Kraimer, & Liden, 2001). In addition, Luthans (1988) asserts that managers’ ability to build networks is the strongest predictor of managerial success that benefits both individuals and organizations. McGuire (2000) states that formal networks are public, official, and have clear boundaries, whereas informal networks are private, voluntary, and characterized by fluid boundaries. Employers usually formally recognize formal work networks, focus on achieving the goals of members and social organizations, and tend to have identifiable membership and network structures ( McGuire 2000). By contrast, participation in informal networks is not formally regulated, and the purpose of such networks may be work-related, personal, or social ( Ibarra, 1992). The formation of networks by university leaders is expected to increase the internationalization activities of higher education by easily obtaining various information and other resources from outside the organization.

This generation of leadership encourages universities to compete globally ( Angreani & Vijaya, 2017). Indicators of success in achieving competitive universities are reflected in the achievement of positions in international rankings/ *World Class Universities* ( Polyakov et al., 2020). The importance of leadership towards the WCU in a university setting is reflected in the expectations of national and international accreditation bodies. Each includes criteria and policies related to leadership ( Burns & Mooney, 2018). Therefore, WCU leaders must have global networking, in which they work in a complex, fast-changing, and often ambiguous international environment. WCU leaders engage in foreign markets, strategize internationally, and manage and motivate teams to compete globally ( Hassan et al., 2011).

### Leadership style

Higher education leaders in the era of neoliberalism not only play academic leaders but also have two roles simultaneously, namely as academic leaders and chief executive officers at the universities they lead ( Ekman, Lindgren, & Packendorff, 2018; Nizam, 2022). Leadership plays an important role in determining the success of higher education ( Osseo-Asare et al., 2005). This is reflected in the expectations of Accreditation Bodies (National and International), including the criteria and policies related to effective leadership in higher education ( Mooney, Burns, & Chadwick, 2012).

The success of a change depends on the leader’s leadership style ( Baesu & Bejinaru, 2014). Leadership style has been recognized as an important aspect of influencing innovation because leaders effectively have an important role in producing ideas, goal setting, and creating organizational culture ( Elrehail, Emeagwali, Alsaad, &; Alzghoul, 2018). In addition, leadership and behavior can build a climate of trust that encourages innovation throughout the organization ( Al-Husseini & Elbeltagi, 2014). To manage these changes, leaders must understand the change management process and demonstrate an appropriate leadership style ( Akinbode &; Shuhumi, 2018; Mansaray, 2019; McRoy & Gibb, 2009). Transformational leadership is believed to be a key element of change-oriented leadership ( Akinbode &; Shuhumi, 2018; Appelbaum et al., 2015), and to understand higher education governance in a period of transformation into the priority of a change leader ( Aldulaimi &; Abdeldayem, 2020).

## Conclusions

After conducting a bibliometric analysis of 662 documents, we discovered that most documents (446) were articles, with the remainder coming from books, chapters, conference papers, editorials, notes, and reviews. The journals that made the most significant contributions to the development of collegiality, change management, and neoliberalism in Higher Education are International Journal of Educational Management (15 documents), Journal of Higher Education Policy and Management (11 documents), Tertiary Education and Management (10 documents), Higher Education (9 documents), Innovations in Education and Teaching International (9 documents), and Journal of Organizational Change Management (9 documents).

Furthermore, there has been a steady increase in articles published annually, with notable spikes in 2017 and 2018 regarding collegiality, change management, and neoliberalism in higher education. The top contributors to publications on collegiality, change management, and neoliberalism in higher education are institutions: Monash University (27 documents), University of The Sunshine Coast (18 documents), University of Massachusetts (10 documents), Curtin University (9 documents), Edinburgh Napier University (nine documents), Kennesaw State University (eight documents), University of Oulu (eight documents), Macquarie University (seven documents), National University of Science and Technology (Misis) (seven documents), and Texas A and M University (seven documents). Institutions from Australia and America still dominate most affiliations related to the relationship between these factors in higher education, while authors from the United Kingdom, the USA, and Australia dominate correspondence institutions. From the publications, it can be concluded that the authors have the most significant relevance in a row, namely, Lazano R. and Tapper T. (six documents), Amaral A., Carvalho T., and Palfreyman D. (five documents), Angehrn AA, Maxwell K. (four documents), Ameen K, Awais S., and Blackmore J. (three documents). The keywords in the word cloud regarding collegiality, change management, and neoliberalism in higher education are organization and management, leadership, curriculum, organizational innovation, organizational change, decision-making, and humans, indicating that the relationship between these concepts is relevant to leadership, organizational change, management, and human resources. The article also added eight issues that can be considered in instituting changes in higher education related to adopting neoliberalism in higher education. How far and how much corrosive impact is created by the adoption of the neoliberal environment, of course, should not rule out the prominent role that is the common goal of higher education, which is to carry out the social function of higher education to create the welfare of the nation, through the development of science and technology and producing intellectuals, scientists, or highly competitive professional human resources.

Implication in the light of the theoretical framework, the study leverages Lewin’s change management model, which includes the stages of unfreezing, changing, and refreezing, to explore the shifts in higher education driven by neoliberalism. This model helps frame how universities can adapt while balancing traditional academic values with new market-driven imperatives, thus managing change while retaining core academic values. This study highlights the shift towards entrepreneurialism and commercialization in higher education institutions, emphasizing revenue generation, corporate partnerships, and market-driven policies. Practical implications involve adopting business-like models, enhancing student satisfaction, and aligning services with stakeholders’ needs. Universities are encouraged to foster collaborations and use digital solutions to maintain global competitiveness and meet academic and financial goals.

### Limitations and future research directions

This study is limited to case studies and critical analyses of literature. There may be a risk of publication bias because the study only included published articles indexed in Scopus, potentially overlooking non-indexed research that could provide different perspectives. Further research is necessary for testing the robustness of the findings to different scenarios, such as excluding studies at high risk of bias or adjusting for publication bias, we can assess the reliability and validity of the synthesized results in the presence of reporting bias.

Further research can be conducted to empirically examine the factors that significantly influence change in universities in the face of the influence of neoliberalism and to examine the extent to which the perspective of university leaders and management considers these factors important for increasing the competitiveness of universities. Although the study results have been presented in this way, there are limitations to revealing various things that may be related beyond the eight issues the authors report. Future research can delve deeper into other factors that influence the implementation of neoliberalism in higher education. Future research could also empirically examine to what extent the influence of these eight issues affects the policy patterns of a change leader in higher education. The future research could conduct GRADE (Grading of Recommendations Assessment, Development and Evaluation) approach for assessing the certainty of evidence and grading the strength of recommendations in systematic reviews. This method provides a transparent and structured framework for evaluating the quality of evidence and informing decision-making. The upcoming research may also conduct the effect measure on performance metrics to assess the alignment of educational services with stakeholder expectations before and after the impact of neoliberal policies on higher education institutions. This effect measure provides a framework for evaluating the impact of neoliberalism on collegiality, governance, management, and quality management methods within the context of higher education institutions.

## Data Availability

Zenodo: Explicating collegiality and change management in neoliberalism during the dynamics of higher education institutions. https://doi.org/10.5281/zenodo.10730018 ( Marlia et al., 2024). This project contains the following underlying data:
- Scopus.csv - Scopus.ris Zenodo: Explicating collegiality and change management in neoliberalism during the dynamics of higher education institutions. https://doi.org/10.5281/zenodo.10730018 ( Marlia et al., 2024). This project contains the following extended data:
-
Figure 1-Concept Map of Changes in Universities.png -
Figure 2-Primary Information.png -
Figure 3-Annual Scientific Production.png -
Figure 4-Average Article Citation per Year.png -
Figure 5-Most relevant sources.png -
Figure 6-Sources’ local impact by H-index.png -
Figure 7-Most relevant authors.png -
Figure 8-Authors’ production over time.png -
Figure 9-Most relevant affiliations.png -
Figure 10-Corresponding author’s countries.png -
Figure 11-Co-occurance network.png -
Figure 12-Word cloud.png -
Figure 13-Tree map.png -
Figure 14-Comprehensive change patterns.png PRISMA Checklist for ‘Explicating collegiality and change management in neoliberalism during the dynamics of higher education institutions’ https://doi.org/10.5281/zenodo.10730018 ( Marlia et al., 2024). Data are available under the terms of the Creative Commons Attribution 4.0 International license (CC-BY 4.0).
