# Supplementary material for: Explicating collegiality and change management in neoliberalism during the dynamics of higher education institutions: A Systematic Literature Review using PRISMA Checklist
Source: F1000Res. 2025 Dec 17;13:237. Originally published 2024 Mar 28. [Version 5] doi: 10.12688/f1000research.146044.5 (PMC12715403; doi:10.12688/f1000research.146044.5)
Supplement: Peer review history

# Reviewer report: Reviewer response for version 5

**DOI:** 10.5256/f1000research.191474.r442448

**Published:** 2026-01-09

**Authors:** Labrina Gioti

**Affiliations:** 1 Aristotle University of Thessaloniki, Thessaloniki, Greece

The revisions of the article are sufficient and covered all the parts and comments of reviewing. It can be indexed as it is.

Are the rationale for, and objectives of, the Systematic Review clearly stated?

Yes

Is the statistical analysis and its interpretation appropriate?

Yes

If this is a Living Systematic Review, is the ‘living’ method appropriate and is the search schedule clearly defined and justified? (‘Living Systematic Review’ or a variation of this term should be included in the title.)

Yes

Are sufficient details of the methods and analysis provided to allow replication by others?

Yes

Are the conclusions drawn adequately supported by the results presented in the review?

Yes

Reviewer Expertise:

NA

I confirm that I have read this submission and believe that I have an appropriate level of expertise to confirm that it is of an acceptable scientific standard.

# Reviewer report: Reviewer response for version 5

**DOI:** 10.5256/f1000research.191474.r442443

**Published:** 2025-12-18

**Authors:** António M Magalhães, Amélia Veiga

**Affiliations:** 1 Education Sciences, University of Porto, Porto, Portugal, Portugal; 2 University of Porto, Porto, Portugal

The authors made, indeed, a visible effort to respond to the issues raised in our review.

 No further comments.

Are the rationale for, and objectives of, the Systematic Review clearly stated?

Partly

Is the statistical analysis and its interpretation appropriate?

Not applicable

If this is a Living Systematic Review, is the ‘living’ method appropriate and is the search schedule clearly defined and justified? (‘Living Systematic Review’ or a variation of this term should be included in the title.)

Not applicable

Are sufficient details of the methods and analysis provided to allow replication by others?

Yes

Are the conclusions drawn adequately supported by the results presented in the review?

No

Reviewer Expertise:

Higher Education Studies

We confirm that we have read this submission and believe that we have an appropriate level of expertise to confirm that it is of an acceptable scientific standard.

# Reviewer report: Reviewer response for version 4

**DOI:** 10.5256/f1000research.180902.r419131

**Published:** 2025-10-14

**Authors:** António M Magalhães

**Affiliations:** 1 University of Porto, Porto, Portugal

Based on the assumption that new managerialism in higher education has transformed collegiality and university governance, the paper aims at identifying and analyzing these changes and their impact on universities and academia. It also aims to analyze how institutional governance and leadership are evolving and adapting to this context.

 The theoretical framework underlying the undertaken systematic review suits the purposes of the study. However, one may wonder if the statement on page 5 on the research gap, “The rationale for the review in the context of existing knowledge is to address the gap in understanding how neoliberalism influences collegiality and change management in higher education institutions”, develops as if there is an actual gap. Indeed, many studies and publications have been addressing, since the 1990s, the effects of NPM and neoliberalism on universities and their actors [e.g., the book by Slaughter & Rhoades, (2004), the book M. Kogan, M. Bauer, I. Bleiklie, M. Henkel (eds.) (2006), the book edited by Catherine Paradeise, Emanuela Reale, Ivar Bleiklie, Ewan Ferlie (2009), or the article by Bleiklie, I., Laredo, P. & Sörlin, S. (2007) or the article by Rosemary Deem (2011)]. The authors must clarify this and its implications on the workings of the systematic review they undertook. The authors recognize the choice for Scopus Database as a limitation of their study. Even though the systematic review focused on articles the paper cannot ignore seminal works and research on the development of the topic in academic literature.

 The reviewers strongly advice to convene seminal authors and publications on the topic, as they did with regard to Burton Clark 1999 work on “Creating Entrepreneurial Universities: organizational pathways of transformation” that directly relate to the study the paper presents.

 The paper needs English editing and a careful text revision. To give an example, the sentence on page 15, “The forces of globalization have driven the literature review” it is far from clear. Does it refer to the authors system literature review?

 The Lewin’s theoretical framework appears as adequate to identify patterns of change. However, the conclusions of the study undertaken do not add to what is already studied, researched and known.

 António Magalhães and Amélia Veiga

Are the rationale for, and objectives of, the Systematic Review clearly stated?

Partly

Is the statistical analysis and its interpretation appropriate?

Not applicable

If this is a Living Systematic Review, is the ‘living’ method appropriate and is the search schedule clearly defined and justified? (‘Living Systematic Review’ or a variation of this term should be included in the title.)

Not applicable

Are sufficient details of the methods and analysis provided to allow replication by others?

Yes

Are the conclusions drawn adequately supported by the results presented in the review?

No

Reviewer Expertise:

Higher Education Studies

I confirm that I have read this submission and believe that I have an appropriate level of expertise to state that I do not consider it to be of an acceptable scientific standard, for reasons outlined above.

# Response

**Published:** 2025-11-13

**Authors:** Makna Ani Marlia

**Affiliations:** Management, Universitas Andalas, Padang, West Sumatra, Indonesia

Dear Prof. António M Magalhães

 We would like to express our sincere appreciation to you and the reviewers for the valuable time and thoughtful feedback provided on our manuscript. The comments have been extremely helpful in improving the clarity, depth, and overall quality of our paper.

 In this revised version, we have carefully addressed each comment and made corresponding revisions throughout the manuscript.

**Reviewer**

 1. Based on the assumption that new managerialism in higher education has transformed collegiality and university governance, the paper aims at identifying and analyzing these changes and their impact on universities and academia. It also aims to analyze how institutional governance and leadership are evolving and adapting to this context.

**Our response:**

 Thank you very much for your thoughtful and constructive feedback. We truly appreciate the time and attention you have given to reviewing our paper. Your insightful comments have been invaluable in helping us strengthen the clarity and depth of our analysis.

 ----

 2. The theoretical framework underlying the undertaken systematic review suits the purposes of the study. However, one may wonder if the statement on page 5 on the research gap, “The rationale for the review in the context of existing knowledge is to address the gap in understanding how neoliberalism influences collegiality and change management in higher education institutions”, develops as if there is an actual gap. Indeed, many studies and publications have been addressing, since the 1990s, the effects of NPM and neoliberalism on universities and their actors [e.g., the book by Slaughter & Rhoades, (2004), the book M. Kogan, M. Bauer, I. Bleiklie, M. Henkel (eds.) (2006), the book edited by Catherine Paradeise, Emanuela Reale, Ivar Bleiklie, Ewan Ferlie (2009), or the article by Bleiklie, I., Laredo, P. & Sörlin, S. (2007) or the article by Rosemary Deem (2011)]. The authors must clarify this and its implications on the workings of the systematic review they undertook. The authors recognize the choice for Scopus Database as a limitation of their study. Even though the systematic review focused on articles the paper cannot ignore seminal works and research on the development of the topic in academic literature.

**Our response:**

 Thank you for your valuable comment. We have revised the paragraph on page 5 to acknowledge earlier seminal works and clarify that the study addresses a methodological, rather than theoretical, gap through a systematic and bibliometric approach. Additionally, we have updated the Limitations and future research directions section (page 36) to explicitly note the exclusion of non-indexed or seminal works and to suggest expanding future research to include them.

 ----

 3. The reviewers strongly advice to convene seminal authors and publications on the topic, as they did with regard to Burton Clark 1999 work on “Creating Entrepreneurial Universities: organizational pathways of transformation” that directly relate to the study the paper presents.

**Our response:**

 Thank you for the constructive suggestion. We have added a new paragraph on page 5 acknowledging Burton R. Clark’s (1998) seminal work Creating Entrepreneurial Universities: Organizational Pathways of Transformation. This addition highlights Clark’s contribution to understanding how universities transform into entrepreneurial institutions under neoliberal pressures. We also clarified how our study builds upon this foundation by extending his conceptual insights through a systematic and bibliometric review of global research trends.

 ----

 4. The paper needs English editing and a careful text revision. To give an example, the sentence on page 15, “The forces of globalization have driven the literature review” it is far from clear. Does it refer to the authors system literature review?

**Our response:**

 Thank you for pointing this out. We have revised (page 15) to clarify that globalization refers to the external forces driving transformation in higher education, not the process of conducting the systematic review.

 ----

 5. The Lewin’s theoretical framework appears as adequate to identify patterns of change. However, the conclusions of the study undertaken do not add to what is already studied, researched and known.

**Our response:**

 Thank you for this valuable feedback. We have revised the Conclusions section to clearly highlight the study’s original contribution beyond previous literature. The updated version emphasizes how the integration of bibliometric and systematic review methods provides new empirical insights into the evolution of collegiality, change management, and neoliberalism in higher education. It also clarifies that the study extends Lewin’s change management framework by demonstrating how its stages, unfreezing, moving, and refreezing manifest within contemporary university contexts influenced by neoliberal ideology. This revision strengthens the theoretical and practical significance of the findings.

# Reviewer report: Reviewer response for version 4

**DOI:** 10.5256/f1000research.180902.r388833

**Published:** 2025-06-03

**Authors:** Maria Alzira Pimenta Dinis

**Affiliations:** 1 Universidade Fernando Pessoa, Porto, Portugal

It is unclear in the title the specific context, if worldwide or focusing in a specific reality.

 Unclear content needs to be revised: “We selected all retrieved sources based on the keywords and analyzed all the documents we obtained.”

 This study did this, this study did that, needs revision.

 General, I would say:

 “The findings show that organizational management, leadership, education, technology, curriculum, innovation, organizational change, decision-making, and human beings are significant trajectories of neoliberalism in higher education.”

 “This study offers other constructs for accelerating leadership success in higher education. This relates to how change leadership can navigate changes resulting from neoliberal ideology.”

 Avoid abbreviations in keywords:

 “Keywords change management, collegiality, neoliberalism, NPM, managerialism, higher education”

 The manuscript need to be revised in terms of flow, interconnecting paragraphs, and removing older references unless absolutely necessary. Language needs overall refinement, as such terminology is perfectly unclear: “lamented”. What government, all governments...?, unclear arguments... “The government”. Which are? “Various phenomena” So, this type of unclear discourse becomes dangerous.

 Low quality DPI figures, not sure if adapted and unclear caption.

 “Figure 1. Concept Map of Changes in Universities (Lewin, 1951).”

 All captions need revision to be made self-explanatory.

 We cannot have duplicated captions:

 “Figure 2. Primary information. Table 1. Primary information.”

 I also do not understand terms such as results in Table 1 or commas in the values.

 Every abbreviation/parameters needs definition under tables, in notes.

 After having checked the manuscript, it would say it needs global formal check, language check, entire streamlining, the text is broken into pieces. Terminology such as “documents” need revision. Why not publications? Then grouped figures require subcaption per letter, after a main caption. The number of Figures is excessive, they should be rearranged or even grouped... I would suggest to avoid “we”

 I am unsure in terms of separating the discussion from results, why not merge? Again, old references and a scattered discourse is a problem. The conclusions section needs to start with a clear context, methods, findings, implications, limitations, further prospects.

 I hope that authors are able to improve the article, accordingly.

Are the rationale for, and objectives of, the Systematic Review clearly stated?

Partly

Is the statistical analysis and its interpretation appropriate?

Partly

If this is a Living Systematic Review, is the ‘living’ method appropriate and is the search schedule clearly defined and justified? (‘Living Systematic Review’ or a variation of this term should be included in the title.)

No

Are sufficient details of the methods and analysis provided to allow replication by others?

Partly

Are the conclusions drawn adequately supported by the results presented in the review?

Partly

Reviewer Expertise:

Sustainability

I confirm that I have read this submission and believe that I have an appropriate level of expertise to state that I do not consider it to be of an acceptable scientific standard, for reasons outlined above.

# Response

**Published:** 2025-06-13

**Authors:** Makna Ani Marlia

**Affiliations:** Management, Universitas Andalas, Padang, West Sumatra, Indonesia

Dear Prof. Dinis

 We would like to express our sincere gratitude to the reviewer for their constructive and insightful feedback. We greatly appreciate the time and effort taken to review our manuscript. Regarding the concerns raised about the title, it is acknowledged that it may not have been sufficiently clear in terms of the specific context it addresses. Clarification will be made to ensure the title reflects whether the study is focused globally or on a specific reality.

 In response to the comment on the methodology section, it is understood that the phrase "We selected all retrieved sources based on the keywords and analyzed all the documents we obtained" may lack clarity. A more accurate and transparent description of the method employed will be provided. Additionally, the suggestion to avoid using phrases such as "This study did this, this study did that" is appreciated, and adjustments will be made to improve the academic tone and clarity of the writing.

 We appreciate the feedback on the phrasing of the findings, particularly the sentence: “The findings show that organizational management, leadership, education, technology, curriculum, innovation, organizational change, decision-making, and human beings are significant trajectories of neoliberalism in higher education.” This statement, along with other findings, will be presented more clearly and concisely.

 Regarding the use of abbreviations in the keywords, the suggestion to avoid them is noted, and the keywords will be adjusted for greater clarity and consistency. The feedback on the term "lamented" is also acknowledged, and more precise language will be used to convey the concern raised in the manuscript.

 The feedback on the figures and captions is appreciated, particularly regarding their clarity and quality. The concern about duplicated captions between Figure 2 and Table 1 has been noted, and improvements will be made to ensure that all figures are clear and self-explanatory.

 In terms of the manuscript's structure, the suggestion to merge the Results and Discussion sections to enhance the flow is understood. The findings will be presented in a more cohesive manner, with clear connections to each research question in the discussion.

 Finally, the review of older references and the overall flow of the manuscript is acknowledged. Efforts will be made to streamline the references, and improvements to the language will be implemented to enhance clarity and precision.

 Once again, we thank the reviewer for their thoughtful comments and suggestions, which have significantly contributed to the improvement of our manuscript. We hope the changes made address the concerns raised and enhance the clarity and rigor of our work.

 Regards,

 Makna Ani Marlia

# Reviewer report: Reviewer response for version 4

**DOI:** 10.5256/f1000research.180902.r386027

**Published:** 2025-05-28

**Authors:** Budi Waluyo

**Affiliations:** 1 Walailak University, Thai Buri, Nakhon Si Thammarat, Thailand

Dear Author(s), 

 I have seen significant improvements in the quality of the manuscript. I think I approve this version. If possible, please proofread the article and ensure the alignment of the references with the journal's guidelines.

 Best,

 Reviewer

Are the rationale for, and objectives of, the Systematic Review clearly stated?

Partly

Is the statistical analysis and its interpretation appropriate?

Not applicable

If this is a Living Systematic Review, is the ‘living’ method appropriate and is the search schedule clearly defined and justified? (‘Living Systematic Review’ or a variation of this term should be included in the title.)

No

Are sufficient details of the methods and analysis provided to allow replication by others?

Yes

Are the conclusions drawn adequately supported by the results presented in the review?

Yes

Reviewer Expertise:

Education

I confirm that I have read this submission and believe that I have an appropriate level of expertise to confirm that it is of an acceptable scientific standard.

# Reviewer report: Reviewer response for version 3

**DOI:** 10.5256/f1000research.178003.r374030

**Published:** 2025-04-26

**Authors:** Labrina Gioti

**Affiliations:** 1 Aristotle University of Thessaloniki, Thessaloniki, Greece

Although the article presents interesting research results, the overall development and documentation is deficient. It seems that the text is written more as an essay than a paper. Adjustments need to be made in order to have a more coherent, synthetic and robust text that is more in line with the format of a paper. Based on the reviewers' comments, some important changes have been made, but the overall impression is that the article lacks coherent and consistent linkage and documentation between theory, research questions and discussion of results.

 Specifically, the introduction is lengthy and descriptive. It does not provide sufficiently the problematic and the state of the art of the research purpose until the end of the section. Data from different countries and institutions are not interrelated and commented. I think it is necessary the writers to state how the three research questions are interconnected and related theoretically. The arguments need a more thorough and concise documentation and critique.  Nor it is clear what theoretical background will inform the research. In the section of "Literature review" the change management aspects  and theories are not connected and/or commented upon with the ideology of neoliberalism and the restructurings of the NPM in higher education institutions. 

 In both sections introduction and literature review, as well as in discussion section, the bibliographical references that substantiate the arguments are presented in successive serialized references and not synthetically, while they are often repeated in the text without a clear and organic integration and connection with the main research questions. 

 In the "Methods" section it would be useful for the authors to mention the tools they used, such as Prisma and bias assessment in the design subsection where they mention the method selection.

 The "Results" are well presented while the figures and the visualisations facilitate reader and illustrate sufficiently the research findings.

 The discussion section is well developed and documented. Nevertheless, the findings are not directly related to the first and third research questions. In the section of "Conclusions"  a general summary is given, but the authors' position and critical stance on the subject is missing.

 Finally, a language check is needed for spelling, syntax and punctuation errors (eg "Lewis Change instead of Lewin's", double parentesis as in (( Nasution, Prasojo, Jannah, & Yumitro, 2020)).

Are the rationale for, and objectives of, the Systematic Review clearly stated?

Yes

Is the statistical analysis and its interpretation appropriate?

Yes

If this is a Living Systematic Review, is the ‘living’ method appropriate and is the search schedule clearly defined and justified? (‘Living Systematic Review’ or a variation of this term should be included in the title.)

Yes

Are sufficient details of the methods and analysis provided to allow replication by others?

Yes

Are the conclusions drawn adequately supported by the results presented in the review?

Yes

Reviewer Expertise:

Adult and Higher Education

I confirm that I have read this submission and believe that I have an appropriate level of expertise to confirm that it is of an acceptable scientific standard, however I have significant reservations, as outlined above.

# Response

**Published:** 2025-05-16

**Authors:** Makna Ani Marlia

**Affiliations:** Management, Universitas Andalas, Padang, West Sumatra, Indonesia

Dear Prof. Labrina Gioti,

 We sincerely thank you for your thorough and constructive feedback on our manuscript "Explicating collegiality and change management in neoliberalism during the dynamics of higher education institutions: A Systematic Literature Review using PRISMA Checklist." Your insights have provided valuable guidance for improving the quality and rigor of our paper. We acknowledge the limitations you have identified and are committed to addressing each point comprehensively in our revision.

- Structure and Academic Format

We appreciate your observation that the manuscript reads more like an essay than a research paper. We will restructure the entire document to better align with standard academic paper conventions, ensuring a more coherent, synthetic, and robust presentation of our research.

- Theoretical Framework and Research Questions

We acknowledge the need to establish clearer theoretical interconnections between our three research questions. The revised introduction will provide a more focused articulation of the research problem and state of the art earlier in the text. We will explicitly demonstrate how the research questions are theoretically interconnected and establish a more robust theoretical foundation that informs our research approach.

- Integration of Literature

We recognize the issue of presenting references in serial listings rather than synthetic integration. Our revised literature review will organize sources thematically and demonstrate critical engagement with the literature. We will ensure that change management theories are explicitly connected to neoliberalism and New Public Management in higher education contexts, creating more organic linkages to our research questions.

- Methods Documentation

We will enhance the Methods section by incorporating details about our use of the PRISMA Checklist and bias assessment tools in the design subsection, with clearer explanations of their implementation. This will provide greater transparency about our methodological approach and facilitate replication by other researchers.

- Discussion and Conclusions

We acknowledge that our findings are not adequately connected to the first and third research questions. The revised discussion section will explicitly address all three research questions, ensuring more direct connections between our results and research objectives. Our conclusion will be strengthened to include our critical stance on the subject and more thoroughly demonstrate how our conclusions are supported by the research findings.

- Technical Issues

We will conduct a comprehensive language review to address spelling, syntax, and citation formatting issues throughout the manuscript, including correcting errors such as "Lewis Change" to "Lewin's Change" and fixing double parentheses in citations.

- Implementation Plan

We are preparing a comprehensive revision that addresses all the points raised in your review. The revised manuscript will feature:

1. A more concise, focused introduction that clearly establishes the research problem and theoretical framework
2. A restructured literature review with thematic organization and clearer connections between change management theories, neoliberalism, and higher education governance
3. Enhanced methodological documentation
4. A more cohesive results and discussion section that directly addresses our research questions
5. A strengthened conclusion that presents our critical stance and implications
6. Thoroughly edited language for clarity and correctness

We believe these revisions will significantly strengthen our manuscript and address the valuable feedback you have provided. We are grateful for your insights, which will help us develop a more rigorous and valuable contribution to the literature on higher education governance in the context of neoliberalism.

 Sincerely,

 Makna Ani Marlia

# Reviewer report: Reviewer response for version 3

**DOI:** 10.5256/f1000research.178003.r369956

**Published:** 2025-03-18

**Authors:** Budi Waluyo

**Affiliations:** 1 Walailak University, Thai Buri, Nakhon Si Thammarat, Thailand

Dear Author(s), 

 Thank you for revising the manuscript. I only have a minor revision to request:

 In the abstract (Methods), you should mention the number of the reviewed articles and where you took them from (databases). You should not include the search keywords. 

 Thank you.

 Reviewer

Are the rationale for, and objectives of, the Systematic Review clearly stated?

Partly

Is the statistical analysis and its interpretation appropriate?

Not applicable

If this is a Living Systematic Review, is the ‘living’ method appropriate and is the search schedule clearly defined and justified? (‘Living Systematic Review’ or a variation of this term should be included in the title.)

No

Are sufficient details of the methods and analysis provided to allow replication by others?

Yes

Are the conclusions drawn adequately supported by the results presented in the review?

Yes

Reviewer Expertise:

Education

I confirm that I have read this submission and believe that I have an appropriate level of expertise to confirm that it is of an acceptable scientific standard.

# Reviewer report: Reviewer response for version 2

**DOI:** 10.5256/f1000research.174074.r343718

**Published:** 2024-12-13

**Authors:** Budi Waluyo

**Affiliations:** 1 Walailak University, Thai Buri, Nakhon Si Thammarat, Thailand

Dear Author(s),

 Thank you for submitting the revised manuscript. Overall, the quality has improved significantly. However, I have some additional comments to further enhance your manuscript.

 First, I recommend revising the background section in the abstract. You should avoid using "THIS" repeatedly throughout the three sentences. Please revise the three sentences and combine them into two, directly stating the research gaps and the contribution of your study.

 Second, in the introduction, could you add some contextual details from the studies you mentioned? For example, you could include information on the countries where the studies were conducted, the participants involved, and the universities or institutions included. This additional context will help scaffold knowledge in readers' minds and emphasize the critical need for your study.

 Third, the first sentence of your literature review ("Change is identified as a shift in organizational behavior from one form to another") is unclear and needs further clarification. Also, you should provide references for this statement. The first paragraph of the literature review lacks coherence and critical engagement. Please revise it accordingly. Furthermore, I suggest adding more references to this section. Reflect on the key terms in your research questions and ensure that these terms are elaborated upon in your literature review, as they will serve as the theoretical or conceptual foundations of your study.

 Fourth, the methods section is adequate, and I am satisfied with it.

 Fifth, the results section appears interesting, and the visualizations are helpful. Nonetheless, I recommend presenting the results in the order of the research questions. Using the research questions as a guide will make the results easier to follow and understand.

 Sixth, the discussion section is adequate; however, as I mentioned earlier, you need to provide more contextual details. You could mention the countries, participants, types of higher education, etc., examined in the prior research you cited. Expanding on the context in the discussion will help broaden the interpretation of your findings.

 Thank you for your hard work on this revision. I look forward to seeing the updated manuscript.

 Best regards,

 Reviewer

Are the rationale for, and objectives of, the Systematic Review clearly stated?

Partly

Is the statistical analysis and its interpretation appropriate?

Not applicable

If this is a Living Systematic Review, is the ‘living’ method appropriate and is the search schedule clearly defined and justified? (‘Living Systematic Review’ or a variation of this term should be included in the title.)

No

Are sufficient details of the methods and analysis provided to allow replication by others?

Yes

Are the conclusions drawn adequately supported by the results presented in the review?

Yes

Reviewer Expertise:

Education

I confirm that I have read this submission and believe that I have an appropriate level of expertise to state that I do not consider it to be of an acceptable scientific standard, for reasons outlined above.

# Response

**Published:** 2025-02-06

**Authors:** Makna Ani Marlia

**Affiliations:** Management, Universitas Andalas, Padang, West Sumatra, Indonesia

Dear Reviewer,

 Thank you for your constructive feedback and for acknowledging the improvements made to our manuscript. We appreciate your detailed suggestions and have addressed each point to further enhance the quality of our work. Below, we outline how we have revised the manuscript in response to your comments.

**Response to Comment 1: Revision of the Background Section in the Abstract** We have revised the abstract to avoid repetitive use of "THIS" and combined the sentences to succinctly state the research gaps and contributions of our study.

**Response to Comment 2: Contextual Details in the Introduction** We have incorporated detailed contextual information from the studies mentioned in the introduction. This includes the countries where the studies were conducted, the participants involved, and the specific universities or institutions examined. This enhancement not only enriches the background but also emphasizes the global relevance and critical need for our investigation.

**Response to Comment 3: Clarification in Literature Review** We have clarified the first sentence of the literature review and provided additional references to support our statements.

**Response to Comment 4: Methods Section** We are pleased to hear that the methods section meets your expectations, and no further revisions have been made here.

**Response to Comment 5: Structure of the Results Section** In response to your suggestion, we have added explain in first paragraph.

**Response to Comment 6: Expanding Contextual Details in the Discussion** We have expanded the discussion section

 We hope these revisions adequately address your concerns and enhance the manuscript's contribution to the literature on higher education under neoliberal influence. We look forward to your feedback and are optimistic about the suitability of our manuscript for publication.

 Thank you once again for your valuable input.

# Reviewer report: Reviewer response for version 1

**DOI:** 10.5256/f1000research.160080.r307169

**Published:** 2024-08-26

**Authors:** Javad Pourkarimi

**Affiliations:** 1 University of Tehran, Tehran, Tehran Province, Iran

Thanks to the scientific efforts of the authors;

 It seems that the researcher should pay attention to more details both in the aspect of problem analysis and in the aspect of methodology, which I will raise two points below:

 The researcher should clearly show what was the existing problem based on which he designed the research. Examine and display the relevant evidence carefully and design it as a scientific research to be able to present the argument well.

 To conduct a systematic study, the researcher must show that enough research has been done in the desired field and the researcher intends to systematically review those studies; Provide a different and creative representation. In addition, there are various types of systematic review that the researcher can refer to the following address- [1]

Are the rationale for, and objectives of, the Systematic Review clearly stated?

Partly

Is the statistical analysis and its interpretation appropriate?

Partly

If this is a Living Systematic Review, is the ‘living’ method appropriate and is the search schedule clearly defined and justified? (‘Living Systematic Review’ or a variation of this term should be included in the title.)

No

Are sufficient details of the methods and analysis provided to allow replication by others?

No

Are the conclusions drawn adequately supported by the results presented in the review?

No

Reviewer Expertise:

Educational Management

I confirm that I have read this submission and believe that I have an appropriate level of expertise to state that I do not consider it to be of an acceptable scientific standard, for reasons outlined above.

## References

[1] : A typology of reviews: an analysis of 14 review types and associated methodologies. *Health Info Libr J* .2009;26(2) : 10.1111/j.1471-1842.2009.00848.x 91-108 10.1111/j.1471-1842.2009.00848.x 19490148

# Response

**Published:** 2024-10-27

**Authors:** Makna Ani Marlia

**Affiliations:** Management, Universitas Andalas, Padang, West Sumatra, Indonesia

We selected PRISMA for our systematic literature review because systematic reviews can report methods and results in sufficient detail to allow users to assess the trustworthiness and applicability of the review findings. We changed the title of this paper.

# Response

**Published:** 2025-04-23

**Authors:** Makna Ani Marlia

**Affiliations:** Management, Universitas Andalas, Padang, West Sumatra, Indonesia

Dear Prof Javad Pourkarimi.

 Thank you for your detailed and constructive feedback. We appreciate your thoughtful review and valuable insights. In response to your feedback:

**Clarification of Research Problem**: We have explicitly clarified the research problem in the introduction, detailing the issues of neoliberalism and its impact on collegiality and change management in higher education. We also carefully examined and presented the relevant evidence supporting the study's design.

**Systematic Study Design**: We have refined the methodology section to clearly outline that we conducted a systematic review based on comprehensive research in the field. The PRISMA methodology is now fully described, and we have included more details on the selection criteria and data analysis process.

**Methodology Transparency**: We have provided a more explicit description of the PRISMA approach and the process by which the studies were selected for review. We also clarified the statistical analysis, emphasizing its appropriateness and relevance to the research objectives.

**Living Systematic Review**: Since our study is not a Living Systematic Review, we removed any references to this method in the revised manuscript.

**Conclusion Support**: We revised the conclusions to ensure they are adequately supported by the results presented in the review. The implications for both theory and practice are now more clearly stated.

 We hope these revisions meet your expectations and improve the clarity and robustness of the manuscript.

 Thank you for your constructive feedback. We look forward to your final approval.

# Reviewer report: Reviewer response for version 1

**DOI:** 10.5256/f1000research.160080.r281536

**Published:** 2024-06-18

**Authors:** Elias Said-Hung

**Affiliations:** 1 International University of La Rioja, Logroño, La Rioja, Spain

It is an interesting work, but it lacks adjustments that could help improve its exposition.

 First of all, I consider that the abstract of the work needs to be reviewed and better structured. The methodology, objectives, results, and relevant conclusions should be more precise.

 Secondly, the work's title should be reviewed, making it clear that it is a literature review.

 Thirdly, the location of the research question should be reviewed. I find it strange that it is stated between the introduction and the literature review. It makes more sense for it to be part of the final paragraph of the introduction section.

 Fourthly, the study should review its general objective to make it more straightforward regarding the type of research carried out. The paper focuses on describing the changes in universities owing to the ideology of neoliberalism through the systematic review, but the results aim to better understand how, from where, and in what academic spaces this topic has been addressed. Something that is not the same. The specific objectives of the study would also need to be made clear so that the presentation of the data has a more coherent narrative, which helps to achieve the general objective finally proposed. Thus, it is better to understand the impact of showing the h-index of the journals where these works are published, for example. Honestly, I consider that the objective of this work focuses more on understanding the frontiers of knowledge reached around the proposed topic than what is proposed as a general objective. Something that can be useful to identify dominant conceptual and methodological approaches better and identify strengths and weaknesses within the academic debate around the topic raised, which helps the development of new works.

 Fifthly, the methodological framework should be better reviewed. The type of methodology considered for the final sample analysis selection should be made clear. In addition to better understanding the selection criteria taken. I understand that a Prisma or similar methodology was applied, but it is something that is not clear. The lack of clarity of specific objectives contributes to the methodology not being transparent about the central categories considered and the convenience of the data described in the results section.

 Sixth, the discussion should be reviewed, considering all the recommendations given. The conclusion section is missing. The discussion and conclusions should clarify the data's implications in light of the theoretical framework, practical applications, limitations, and future lines of work.

Are the rationale for, and objectives of, the Systematic Review clearly stated?

Partly

Is the statistical analysis and its interpretation appropriate?

Partly

If this is a Living Systematic Review, is the ‘living’ method appropriate and is the search schedule clearly defined and justified? (‘Living Systematic Review’ or a variation of this term should be included in the title.)

Yes

Are sufficient details of the methods and analysis provided to allow replication by others?

Partly

Are the conclusions drawn adequately supported by the results presented in the review?

Partly

Reviewer Expertise:

My research areas are in the sociology of education, sociology of communication, hate speech, disinformation content, and polarization. Most of my research is based on quantity analysis and systematic reviews, amongst others.

I confirm that I have read this submission and believe that I have an appropriate level of expertise to state that I do not consider it to be of an acceptable scientific standard, for reasons outlined above.

# Response

**Published:** 2024-10-27

**Authors:** Makna Ani Marlia

**Affiliations:** Management, Universitas Andalas, Padang, West Sumatra, Indonesia

The abstract's structure is based on the author’s guidelines.  Thanks for correcting our title; we revised the title. The position of the research questions is placed in the last paragraph of the Introduction. The study's specific objective is to explore and clarify the role of collegiality and change management within the context of neoliberalism in higher education institutions. This objective is achieved through a systematic literature review that examines how these institutions navigate and adapt to dynamic environments influenced by neoliberal policies. We added implications in the light of theoretical framework and practical application at the end of the conclusion section. We made the limitations and future research direction stand-alone after the conclusion section.

# Response

**Published:** 2025-04-23

**Authors:** Makna Ani Marlia

**Affiliations:** Management, Universitas Andalas, Padang, West Sumatra, Indonesia

Dear Prof. Elias Said-Hung

 Thank you for your thoughtful suggestions. We have addressed your comments as follows:

**Abstract Structure**: We have restructured the abstract to better present the methodology, objectives, results, and conclusions, making it more concise and focused.

**Title Revision**: The title has been revised to clearly indicate that this is a literature review, in line with your suggestion.

**Positioning of Research Questions**: As suggested, we moved the research questions to the last paragraph of the introduction for better flow and clarity.

**General Objective and Specific Objectives**: We have revised the general objective to align more clearly with the systematic review conducted. The specific objectives are now clearly stated, ensuring coherence throughout the manuscript. We also clarified the role of collegiality and change management within the context of neoliberalism in higher education.

**Methodological Framework**: We clarified the methodology section, including the PRISMA framework and the criteria for selecting the studies reviewed. We also ensured that the sampling and analysis methods are transparent and replicable.

**Discussion and Conclusion**: We have expanded the discussion section, incorporating the context and implications of our findings. The conclusion now addresses the theoretical framework, practical applications, limitations, and directions for future research as separate sections.

 We believe these revisions enhance the manuscript's clarity and coherence, and we appreciate your guidance throughout this process.

 We are grateful for the detailed feedback and believe that these revisions significantly strengthen the manuscript. We look forward to your continued feedback and hope the revised manuscript meets your expectations for publication.
